# Amyloid fibrils prepared using an acetylated and methyl amidated peptide model of the α-Synuclein NAC 71–82 amino acid stretch contain an additional cross-β structure also found in prion proteins

**DOI:** 10.1038/s41598-019-52206-5

**Published:** 2019-11-04

**Authors:** Thomas Näsström, Per Ola Andersson, Christian Lejon, Björn C. G. Karlsson

**Affiliations:** 1 Neurodegenerative Disorders Unit, Linnæus University, SE-392 31 Kalmar, Sweden; 2 FOI Swedish Defence Research Agency, CBRN Defence & Security, SE-901 82 Umeå, Sweden; 3 Department of Engineering Sciences: Applied Material Science, Uppsala University, SE-751 21 Uppsala, Sweden; 4 Physical Pharmacy Laboratory, Linnæus University Centre for Biomaterials Chemistry, Linnæus University, SE-392 31 Kalmar, Sweden

**Keywords:** Intrinsically disordered proteins, Diseases of the nervous system

## Abstract

The 71–82 fragment of the non-amyloid-β component (NAC) region of the Parkinson’s disease (PD) and dementia with Lewy bodies (DLB) related protein α-Synuclein, has been reported to be important during protein misfolding. Although reports have demonstrated the importance of this fragment for the aggregation properties of the full-length protein, its exact role in pre-fibrillar oligomerisation, fibrillar growth and morphology has not yet been fully elucidated. Here, we provide evidence that fibrils prepared from an acetylated and methyl amidated peptide of the NAC 71–82 amino acid stretch of α-Synuclein are amyloid and contain, in addition to the cross-β structure detected in the full-length protein fibrils, a cross-β structure previously observed in prion proteins. These results shed light on the aggregation propensity of the NAC 71–82 amino acid stretch of the full-length protein but also the roles of the N- and C-terminal domains of α-Synuclein in balancing this aggregation propensity. The results also suggest that early aggregated forms of the capped NAC 71–82 peptide generated structures were stabilised by an anti-parallel and twisted β-sheet motif. Due to its expected toxicity, this β-sheet motif may be a promising molecular target for the development of therapeutic strategies for PD and DLB.

## Introduction

Neurodegenerative disorders such as Parkinson’s disease (PD) and dementia with Lewy bodies (DLB) are becoming more common probably as life expectancies are increasing. These disorders are associated with neuropathological hallmarks such as loss of neurons in certain parts of the brain accompanied by intracellular deposition of Lewy bodies and Lewy neurites in surviving neurons. The major constituent of Lewy bodies is the misfolded and fibrillated α-Synuclein protein^1^, which has an important part in the pathogenesis of PD and DLB. However, *in vitro* and *in vivo* results suggest that intermediate pre-fibrillar soluble forms, such as oligomers and protofibrils of α-Synuclein, possess more cell degenerating properties than insoluble Lewy body inclusions^2–6^. The exact physiological role of α-Synuclein is not well understood, although reports suggest that it is involved in neurotransmitter regulation^7,8^. The α-Synuclein protein consists of 140 amino acids, is generally natively unfolded, and has lipid binding properties due to four amphipathic and conserved 11-repeats (KTKEGV) of the net positively charged N-terminal region (1–60 aa). Although it is defined as an intrinsically disordered protein, previous structural investigations revealed that α-Synuclein folds into a helical structure, thereby following an apolipoprotein-type A2 helix mechanism, when bound to SDS micelles^9^ and phospholipid vesicles^10^. The binding of misfolded states of α-Synuclein to lipid membranes is believed to be crucial for the development of PD and DLB. The exact binding mechanism is not fully understood, although a recent study suggested that oligomers can create membrane pores^11^ or stabilise pre-existing membrane defects^12^.

The C-terminal (96–140 aa) region of α-Synuclein is rich in acidic residues, which confer a net negative charge and a random coil structure. The central region, also known as the non-amyloid-β component (NAC, 61–95 aa), contains hydrophobic residues that are crucial for protein aggregation^13,14^. Rodriguez *et al*.^15^ recently reported the structural characterisation of generated fibrils prepared from model peptides representing the amino acid stretches 68–78 aa and 69–77 aa. These amino acid stretches belong to the central core of α-Synuclein (NACore, 68–78 aa or the SubNACore, 69–77 aa), and are important for the formation of highly ordered β-sheet-rich structures that are cytotoxic. Detailed investigations revealed that the NACore was more prone to aggregation than the SubNACore, indicating the importance of the amino acid sequence for driving α-Synuclein fibril formation.

A 12 aa stretch of the NAC region residing between 71–82 aa (VTGVTAVAQKTV) is key for the aggregation propensity of the whole NAC fragment. Deletion of this amino acid stretch in the homologous protein β-synuclein leads to an abrogation in the ability to aggregate^16^. A synthetic peptide based on the NAC 71–82 fragment binds to anionic lipid bilayers^17^ and to neutrally charged lipid vesicles^18^. These combined results highlight the significance of studying the aggregation propensity of the NAC 71–82 fragment and the role of this region on the interaction of α-Synuclein with macromolecular targets under normal physiological or pathological conditions.

Previous studies on the aggregation propensity of the NAC 71–82 fragment of α-Synuclein used a model peptide. However, these studies used synthetic peptide preparations with charged N- and C- termini (non-capped) due to the high hydrophobicity of the NAC 71–82 amino acid stretch, thereby mimicking the result from proteolysis of the full-length protein.

Incubation of non-capped NAC 71–82 peptide solutions have resulted in premature amorphous fibrillar aggregates if the solution was incubated for less than six weeks^19^. It is reasonable to assume that introduction of N- and C-terminal charges can generate a peptide that is not a good model for the same amino acid stretch when incorporated into the native full-length protein.

Although the effects of N- and C-termini capping on the aggregation propensity of the NAC 71–82 fragment have not yet been fully elucidated, biophysical and biochemical studies using N- and C- termini capped peptide fragments of the larger Islet Amyloid Polypeptide (IAPP) suggested that neutralisation of charged termini increased aggregation propensity and changed fibril morphology^20^. Therefore, it was deemed important to determine the aggregation propensity and fibril morphology of the NAC 71–82 fragment of α-Synuclein using a capped model peptide representing this amino acid stretch of the full-length protein. The significance of the structures derived from such studies may identify biological motifs of pathological NAC 71–82 fragment aggregates that could be isolated and used for drug development.

In this study, we use data obtained from a series of spectroscopic [attenuated total reflectance Fourier transform infrared (ATR FT-IR) spectroscopy, photon cross correlation spectroscopy (PCCS), and time correlated single photon counting (TCSPC) spectroscopy] and microscopic [transmission electron microscopy (TEM) and Congo red staining] analyses to determine that the capped NAC 71–82 peptide fragment of α-Synuclein readily forms amyloid fibrils that contain an additional cross-β structure previously identified in prion proteins. By contrast, the non-capped NAC 71–82 fragment forms premature amorphous fibrillar structures, in agreement with previous reports. Cluster analysis of the population of structures obtained from molecular dynamics (MD) simulations of the early pre-fibrillar stage of both model peptides, revealed the presence of oligomeric structures containing an antiparallel twisted β-sheet motif in the capped NAC 71–82 peptide fragment. These combined results shed light on the true aggregation propensity of the NAC 71–82 amino acid stretch of the full-length α-Synuclein protein, and demonstrate the potential use of the capped NAC 71–82 peptide as a target for the development of therapeutic strategies for PD and DLB.

## Methods

### Chemicals

Recombinant wild-type α-Synuclein (lyophilised powder in final buffer concentration of 20 mM Tris-HCl pH 7.4 and 0.10 M NaCl) was obtained from rPeptide (>95% purity (SDS PAGE and MS), Watkinsville, GA, USA). The capped NAC 71–82 aa α-Synuclein peptide fragment (VTGVTAVAQKTV) (acetylated N-terminus and methylated amidated C-terminus) was purchased as a lyophilised trifluoroacetate (TFA) salt from Caslo ApS (96.3% purity (HPLC and MS), Kongens Lyngby, Denmark). The non-capped NAC 71–82 aa variant was purchased as a lyophilised TFA salt from GenScript (95% purity (HPLC and MS), Piscataway Township, NJ, USA). All peptide and protein samples were purchased as pre-weighed powders in Eppendorf tubes (1.0 mg α-Synuclein and 4.0 mg peptides).

The colloidal silica (LUDOX®) particles (34 weight-% suspension in deionised water), Thioflavin-T (ThT) (≥65% dye content), the Congo red staining kit (HT-60), and xylene (histological grade) were purchased from Sigma-Aldrich (St. Louis, MO, USA). Vectashield mounting medium was from Vector Laboratories (Burlingame, CA, USA). Ethanol (99.5%) was obtained from Solveco, Rosersberg, Sweden. Millipore (Millipore, Bedford, MA, USA) water was used for all experiments.

### Instruments

Fluorescence steady-state emission spectra were recorded on a SPEX FluoroMax-2 fluorimeter (HORIBA Jobin Yvon, Edison, NJ, USA). Fluorescence decay times were obtained through the time correlated single photon counting (TCSPC) technique using a time-resolved spectrometer equipped with a data station hub, TBX-04 photon detection module, NanoLED (453 nm, 1.0 MHz repetition rate), and a 5000 M fluorescence monochromator (all from IBH Ltd., Glasgow, Scotland). Electron micrographs were acquired with a Talos L120 transmission electron microscope (TEM, Thermo Fisher Scientific, Waltham, MA, USA) equipped with a Ceta CMOS 4 K × 4 K pixel camera. Images were captured using a Nikon Eclipse E400 (Nikon, Minato, Japan) microscope equipped with a first-order red compensation filter for plane polarised light. Peptide fibril particle size distributions in liquid suspensions were determined by photon cross correlation spectroscopy (PCCS) using a NanoPhox instrument (Sympatec GmbH, Germany). Platinum attenuated total reflectance Fourier transform infrared (ATR FT-IR) spectroscopic measurements were performed on a Vertex 70 instrument equipped with a liquid nitrogen-cooled MCT detector (Bruker, Billerica, MA, USA).

### Fibril preparation

Fibrils were prepared *in vitro* as follows. Buffer solution (20 mM Tris-HCl pH 7.3 and 0.15 M NaCl) was added to the pre-weighed lyophilised samples of the non-capped NAC 71–82 peptide and the native α-Synuclein protein. The capped NAC 71–82 peptide had poor solubility in isotonic solvents (determined by the supplier solubility tests and reflecting the peptide hydrophobicity), so Millipore water was added to the sample. The final concentration of all samples was 2 mg · mL^−1^ to give an equimolar concentration of available amino acids for aggregation (140 μM α-Synuclein and a 12-fold higher peptide concentration to reflect the 12-fold higher number of amino acids in the α-Synuclein protein). Subsequently, all samples were treated identically using repeated steps of vortexing and ultrasound sonication. The dissolved peptide preparations were incubated on a shaker (600 rpm) at 37 °C for 72 h. After 72 h, fibril samples were stored at −20 °C until further analysis.

### ATR FT-IR spectroscopy

Initially, 5 μL of each fibril suspension was applied and left to dry at ambient temperature on the ATR diamond surface. A spectral series was recorded between 4000–600 cm^−1^ with an optical resolution of 4 cm^−1^ until the water peak was at a minimum. For each fibril type, the secondary structure components of the Amide I band (1700–1600 cm^−1^) were inspected, and the number of peaks was determined by obtaining the second derivative of each spectra. To characterise initial solid states for peptide and protein preparations before fibrillisation, spectra of the Amide I band were recorded using lyophilised undissolved powder.

### Molecular dynamics (MD) simulations

Single extended structures of the capped NAC 71–82 and the non-capped NAC 71–82 peptides were built using XLEaP, a module of AmberTools15 (v.15, USCF, San Francisco, CA)^21^. Then, 10 copies of each peptide were randomly mixed using PACKMOL^22^ and random seed numbers with 10 000 water molecules and 10 neutralizing chloride ions in cubic boxes with 84-Å sides. For molecular systems including the non-capped NAC 71–82 peptide, additional sodium and chloride ions were included for a final physiological salt concentration of ~0.15 M. To improve statistics for each type of peptide–solvent system, a total of 10 boxes were built and analysed for each system (20 systems in total). Information on the number and type of peptide, number of water molecules and ions included in each box, and the initial and final size of each system (after running MD simulations) is given in supplementary Table S1. The peptide–solvent systems built with PACKMOL^22^ (see previous section) were loaded into the XLEaP module of AmberTools15 (v.15, USCF, San Francisco, CA) together with the Amber14SB force field^23^. The TIP3P-model was used for water, and the Joung and Cheatham^24^ parameters were used for sodium and chloride ions.

All systems were initially energy-minimised, to remove high-energy vdW contacts (5000 steps of steepest descent and 5000 steps of conjugate gradient). In a second step, each system was equilibrated using MD simulation to reach conditions of NVT (constant number of particles, volume, and temperature) increasing the temperature from 0 to 310.15 K for 100 ps. After the target temperature was reached, an additional 500 ps of MD simulation at constant NPT conditions (constant number of particles, pressure, and temperature) was conducted using a target isotropic pressure of 1 bar. During both MD simulation steps, the peptide fragments were restrained with a force constant of 10.0 kcal · mol^−1^ · Å^−2^. Finally, the peptide fragments restraint was released and 1 μs of MD simulation data was collected for each peptide system at NPT conditions (1 bar and 310.15 K) saving data every 10 ps. A total of 10 μs of trajectory data on the folding and aggregation dynamics for each peptide were collected and analysed. All MD simulations were performed using AMBER14 (v.14, UCSF, San Francisco, CA)^21^. A 0.002 ps time-step was used in all simulations, and the SHAKE algorithm^25^ was used to constrain all bonds to hydrogen. A 10-Å cutoff was used for non-bonded interactions, and periodic boundary conditions were employed in all directions. Long-range electrostatic interactions were handled using the Particle Mesh Ewald (PME)^26,27^ summation method, and long-range vdW interactions were treated using a continuum model correction to energy and pressure. Temperature was held constant using the Langevin thermostat with a collision frequency set at 1.0 ps^−1^. Pressure was held constant using the Berendsen barostat and a 2-ps pressure-time relaxation constant.

### Analysis of MD simulation data

Final trajectories were analysed using the CPPTRAJ module implemented in AmberTools15. Radial distribution functions (RDFs) were calculated to quantify water accumulation around the peptide bond of the non-capped and capped peptides. To evaluate the effects of charged termini on water solvation, RDFs were calculated for the NAC 73–80 region of both peptides. The RDF is defined as the ratio between the observed number density of a specified solvent atom at a known distance for a specified solute atom and the average bulk atom number density of the specified solvent atom. RDFs representing the water oxygen atom distribution around the backbone carbonyl oxygen and the backbone amide nitrogen atoms were calculated. The average number of water molecules around the selected peptide backbone functionalities was obtained by integrating the first solvation shell (using a 5.5-Å cutoff) with respect to the resultant RDFs (Supplementary Figs S1–S4 present typical RDFs for the water solvation of the non-capped and capped NAC 71–82 peptides and Figs S5–S8 present computed atomic number densities).

To quantify the secondary structure propensity of each non-capped and capped NAC 71–82 peptide fragment, secondary structure assignments were computed using the dictionary of secondary structure of proteins (DSSP) algorithm^28^ implemented in AmberTools15. The total occupancy of each type of secondary structure element populated during the total MD simulation time was presented as the mean ± standard error of the mean from ten separate MD simulations of 1 μs each (Supplementary Figs S9 and S10).

Cluster analysis was performed on 100 000 structures obtained from each independent MD simulation using the density-based clustering algorithm, DBScan^29^. Root mean square distance (RMSD) of the peptide backbone Cα atoms was used as the distance metric (distance cutoff (epsilon) set to 2.5 Å) and the lowest number of cluster members was set to 30. Top-ranked, highly populated structures derived from DBScan clustering, were visualised in VMD (v.1.9.1, University of Illinois at Urbana-Champaign, USA)^30^ (Supplementary Figs S11 and S12). The secondary structure elements revealed in these geometries were computed using the STRIDE algorithm^31^.

### ThT-fibril binding

The interaction of ThT and the prepared fibrils was analysed by steady-state time-based fluorescence emission spectroscopy. ThT binding to fibrils was monitored by measuring the ThT fluorescence emission over time (λ_exc_ = 450 nm and λ_em_ = 485 nm). Measurements were initiated by equilibrating a fibril solution for 3 min and then adding ThT. The final concentrations of fibril and ThT in each sample were 1.8 mg · mL^−1^ and 10 μM, respectively. ThT fluorescence life time in the presence of fibrils was resolved using TCSPC. ThT fluorescence decay was measured at λ_em_ = 485 nm, which was set by a single grating monochromator with a spectral bandwidth of 16 mm. The excitation source was a NanoLED (IBH) producing 453-nm excitation pulses at a 1.0 MHz repetition rate. During each measurement, the photon-counting rate was always lower than 2% of the excitation source repetition rate to avoid photon pile-up effects. Before every sample measurement, the instrument response function was measured using a LUDOX® solution (excitation and emission wavelength both set to 453 nm). Fluorescence decays were collected over 4096 channels (each channel calibrated to 13.4 ps · channel^−1^), and fluorescence lifetimes were resolved using either a single or a double exponential decay fit model in Analysis Software (v.6.1.51, IBH, Glasgow, Scotland). Generally, curve-fits were accepted when χ^2^ < 1.2. All measurements were performed at ambient temperature under rigorous and continuous stirring, and samples were analysed in quartz Suprasil® cuvettes (3.0-mL and 1-cm path length, Hellma GmbH Müllheim, Germany).

### PCCS

Fibril particle size was determined using PCCS (NanoPhox, Sympatec, GmbH, Germany). Particle size distribution was measured utilising the time auto-correlation functions representing the scattered light generated from analysed particles and assuming Brownian motion. All fibril samples (typically 0.5 mg · mL^−1^) were initially equilibrated at 25 °C in plastic 50-μL UVettes (Eppendorf, Hamburg, Germany), before measurements. To ensure a stable signal (auto correlation), 1-min measurements were sufficient. During each measurement, the refractive index was set to 1.5 with no imaginary part^32,33^. Freshly dissolved control peptide solutions (*i*.*e*., not incubated) did not scatter sufficient light for NanoPhox detection, which was evidence of the lack of particles in those samples. Correlation lengths were evaluated with the auto non-negative least squares (NNLS) with or without a filter to achieve acceptable correlation functions.

### TEM

Samples (3.5 µL protein/peptide dispersion) were drop-coated onto pre-cleaned [glow-discharged with PELCO easiGlow (Ted Pella, Redding, CA, USA)] formvar and carbon-coated copper grids, and subsequently negatively stained with 1.5% uranyl acetate.

### Congo red staining and microscopy

Aggregated preparations of the α-Synuclein protein and capped NAC 71–82 and non-capped NAC 71–82 peptide fragments where stained for amyloids using a Congo red kit according to the manufacturer’s protocol with slight modifications (described below). Stained samples were then examined under brightfield and plane polarised light. Briefly, 2, 10, and 50 μL of 2 mg · mL^−1^ aggregated α-Synuclein protein, capped NAC 71–82 peptide and non-capped NAC 71–82 peptide was added to the centre of microscope slides (Knittel Glass, Braunschweig, Germany) and left to dry at room temperature. In parallel, fresh preparations of alkaline alcoholic (80% v/v) NaCl-saturated solution and Congo red (0.2 weight%) NaCl-saturated solution were prepared and filtered through a 0.2-μm syringe filter. Next, microscope slides were quickly immersed in the 80% (v/v) alcohol NaCl-saturated solution, immediately transferred to alkaline Congo red solution, and then incubated for 20 min at room temperature. The slides were rinsed three times in 99.5% ethanol followed by final immersion in xylene. Vectashield mounting medium was then added to protect the samples before adding cover slips. The slides were examined using a Nikon Eclipse E400 microscope equipped with a first-order red compensation filter for plane polarised light. Images were photographed using a brightfield microscope, and the measured plane polarised light was processed using Nikon NIS Elements software (v.4.13 Nikon, Minato, Japan). Brightfield images were photographed using 20× magnification, 0.25 s exposure time, and 3.40× gain. Polarised images were photographed using 3.40× magnification and 1.5 s exposure time. The colour contrast was set to high and the auto-white option was used. All images were processed with Gimp (v. 2.10.8, www.gimp.org) software. The background of brightfield microscopic images was initially set to white, and the background of the acquired apple-green polarised images was set to black using the levels tool.

## Results and Discussion

Thioflavin-T (ThT)-binding^34^ or ThS-binding, positive Congo red staining with apple-green birefringence, FT-IR detection of β-sheet structure, and TEM analysis of fibrillar morphology are commonly used to detect amyloid structure in aggregation-prone proteins and their ability to form aggregates^35^. Previous studies on amyloidogenic properties of peptides such as amyloid-β (Aβ)^36^ and IAPP^20^, model peptide fragments of full-length proteins such as Apolipoprotein A-IV (ApoA-IV)^37^, and deletion strains of full-length proteins^38,39^ have investigated the effects of charge balance, the role of specific amino acid sequences and the introduction of mutations on aggregation propensity. Madine and co-workers^19^ studied the importance of peptide charge balance on aggregation kinetics by evaluating the effects of pH on aggregation propensity and amyloid structure using the non-capped NAC 71–82 fragment of α-Synuclein. The results showed that the peptide aggregated into β-sheet-rich amyloids at pH 7 and pH 10. At pH 4, the peptide remained as an unfolded structure. In these studies, the authors based their amyloid characterisation on TEM analysis and circular dichroism (CD)-spectroscopy, and the peptide was incubated for 6 weeks. To further characterise the importance of charge balance on fibril formation, we investigated the aggregation propensity of the NAC 71–82 peptide after capping its termini, which was believed to have an effect on final fibril morphology. We present data on the aggregation behaviour of α-Synuclein and models of the NAC 71–82 amino acid stretch of the full-length protein. The peptide concentrations (mg · mL^−1^) were selected to represent the same number of amino acids in the full-length protein. In addition, our approach was used to model the aggregation propensity of the NAC 71–82 amino acid stretch of α-Synuclein upon molecular crowding conditions.

**Figure 1:**
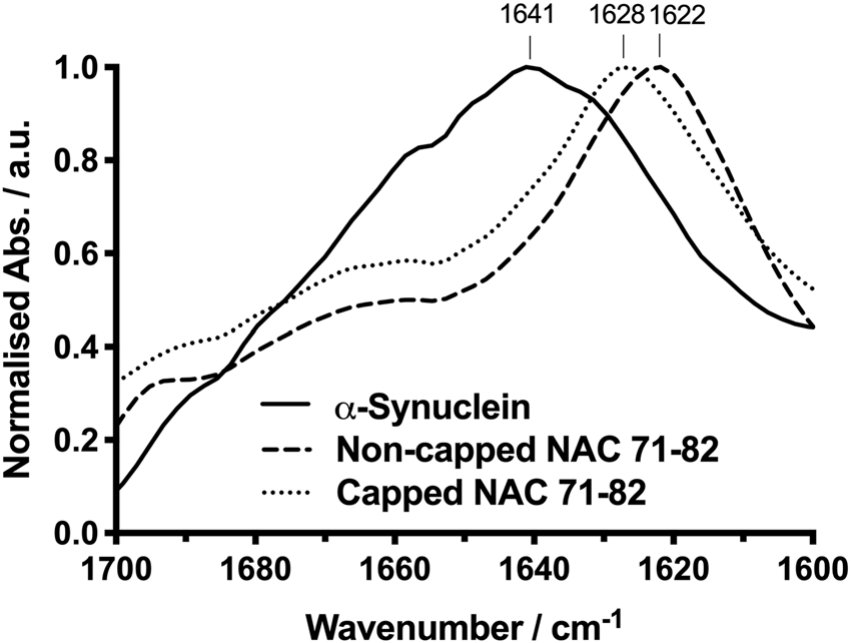
ATR FT-IR solid-state spectra. Shown spectra represent lyophilised α-Synuclein (bold line), non-capped (dashed line) and capped (dotted line) NAC 71–82 peptides in the solid state.

The solid states of α-Synuclein protein and non-capped and capped NAC 71–82 peptides were analysed with ATR FT-IR spectroscopy to ensure sample purity and absence of fibrils. An ATR FT-IR solid-state spectrum of α-Synuclein revealed a broad featureless Amide I vibrational band with a maximum at 1641 cm^−1^ (Fig. 1). This band was associated with random coil (frequency limits 1644.5–1637 cm^−1^)^40^, and was previously reported for monomeric α-Synuclein in solution^41^. The Amide I bands obtained for the non-capped and capped NAC 71–82 peptides demonstrated similar curve profiles and were richer in structure than the band observed in the α-Synuclein spectrum. The observed peaks represented folded structures similar to those reported for synthetic peptides such as polyalanine^42^. The similar solid-state curve profiles for the peptides suggested comparable initial states. The major bands for the non-capped and capped NAC 71–82 peptides were observed at 1622 cm^−1^ and 1628 cm^−1^, respectively. These positions were previously associated with β-sheet structures (1613–1637 cm^−1^). The observed difference in the vibrational maxima (6 cm^−1^) of the non-capped and capped NAC 71–82 peptides was believed to originate in terminal charge difference between the peptides (and possibly peptide orientation), which leads to stronger electrostatic interactions in the non-capped NAC 71–82 peptide in the solid state. The synthetic peptides were prepared as TFA salts, and the main contributing Amide I band from TFA is centred at 1670–1680 cm^−1^. A comparison between the ATR FT-IR spectra of the peptides revealed no evident TFA peak; thus, the amount of TFA present is assumed to be comparable in the peptide samples and thus not interfere in the spectral analysis.

Fibrils were generated by incubating samples (2.0 mg · mL^−1^) for 72 h at 37 °C. Visual inspection of incubated samples revealed precipitates for all preparations. The non-capped NAC 71–82 precipitates were more light scattering and less transparent than the capped NAC 71–82 peptide and α-Synuclein precipitates. As a control, fibrillisation of the non-capped peptide in pure aqueous solution did not produce visual precipitates. This behaviour can be explained by the absence of counter ions capable of minimising repulsion between the terminal charges of interacting peptides, thus reflecting a micellar-like aggregation process. This important role of electrolytes in the extent of fibrillisation is in agreement with the results of Dong *et al*.^41^, who reported faster α-Synuclein fibrillisation kinetics with elevated salt (NaCl) concentrations. These insights suggest different aggregation pathways for the capped and the non-capped NAC 71–82 peptide fragments.

**Figure 2:**
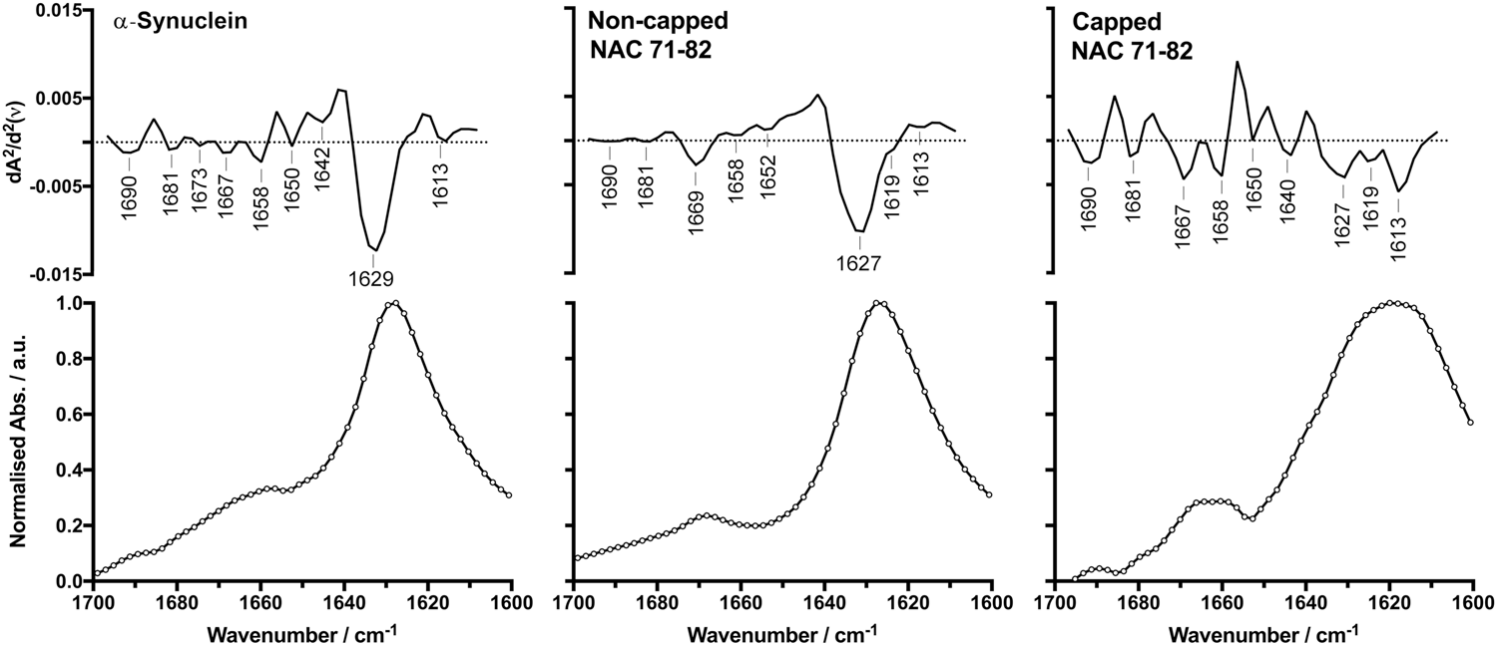
Spectral characterisation of ATR FT-IR data of α-Synuclein and peptides in the fibrillar state. Lower panels show recorded Amide I band spectra of the fibrillar structures (solid lines with open circles) of α-Synuclein protein, non-capped NAC 71–82 peptide, and capped NAC 71–82 peptide. Top panels show processed second-derivative spectra with highlighted wavenumbers representing calculated minima.

ATR FT-IR spectra of the Amide I band of α-Synuclein and the capped and non-capped NAC 71–82 peptides after fibrillisation for 72 h (solid lines with open circles) revealed evident shifts in the secondary structure population from random coil to β-sheet structure (1629 cm^−1^) (Fig. 2). The final position of this vibrational band (1628 cm^−1^) corresponds to a distinct β-sheet structure observed in previous α-Synuclein *in vitro* fibrillisation studies^43^. A change in the position of the vibrational band corresponding to β-sheet structure also was observed for the studied peptides. Peptide fibrillisation increased the intensity of the same β-sheet vibrational band that was observed in the ATR FT-IR spectra for the solid-state peptides (Fig. 1). In some cases, the ATR FT-IR spectrum of a solid-state peptide can differ from that in solution; however, this work focused on end-point fibrillar structure and did not investigate solvent-dependent conformational changes. The second derivative of the Amide I band was computed to deconvolute overlapping vibrational bands and obtain data on the secondary structure of each fibrillar system (Fig. 2). The second-derivative spectra revealed the presence of a similar β-sheet band in all samples (centred at 1627–1629 cm^−1^).

A low-frequency (LF) vibrational band at 1613 cm^−1^ was detected for the capped NAC 71–82 peptide fibrils. This LF band was observed in the FT-IR spectra of other amyloidogenic peptides, and is believed to originate from highly ordered β-sheet structures^44^. The LF band was centred at 1615 cm^−1^ and 1616 cm^−1^ for fibrils of the diabetes type II hIAPP polypeptide^45^ and the lens-associated γD-crystallin protein^46^, respectively. Interestingly, the major β-sheet band for of the prion protein (PrP) was observed at 1626–1628 cm^−1^, whereas the LF band was centred at 1613 cm^−1^ ^47^. Subsequent X-ray crystallography studies indicated that these two bands (designated as CB-1 and CB-2) could be correlated to different cross-β structures^44^. The LF band was attributed to CB-2 and was believed to be a cross-β structure with a different β-strand distance and compactness than that observed for CB-1.

**Figure 3:**
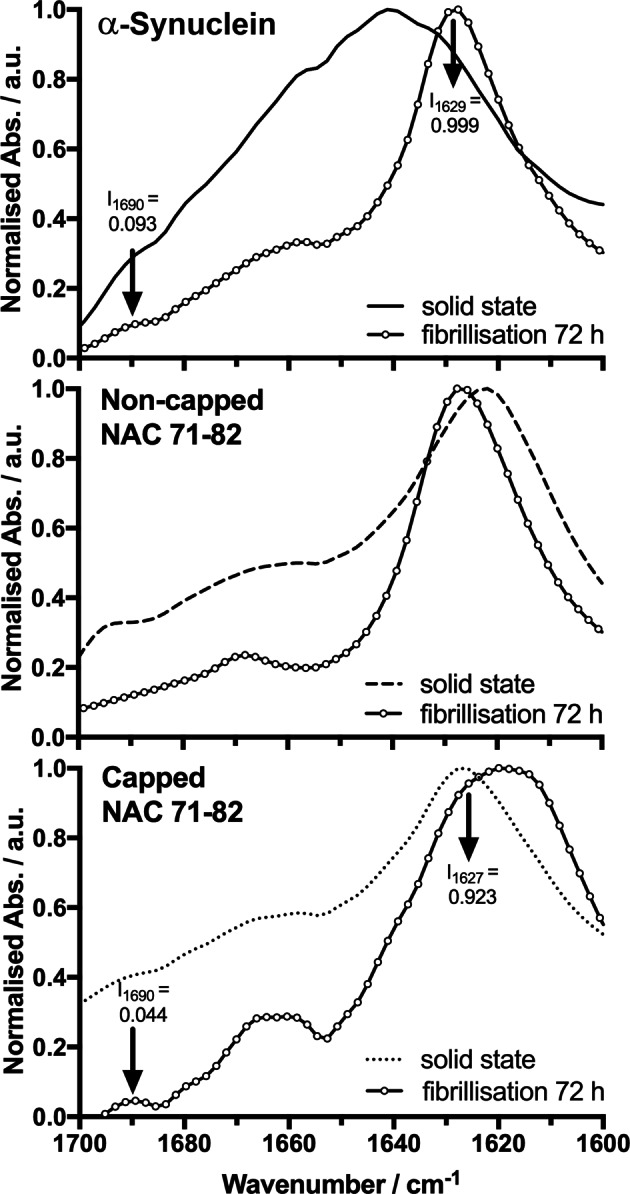
ATR FT-IR spectral comparison between the α-Synuclein protein and peptides in the solid and fibrillar states. Spectral data show the α-Synuclein protein (solid line) and the non-capped NAC 71–82 peptide (dashed line), and capped NAC 71–82 (dotted line) peptide in the solid state and after fibrillisation for 72 h (solid lines with open circles).

Although studies using solid-state NMR (PDB: 2N0A)^48^ and cryo-electron microscopy (PDB: 6A6B)^49^ reported a parallel orientation of β-strands in end-point amyloid structures, vibrational bands in ATR FT-IR spectra associated with anti-parallel β-sheets in fibrillar samples have been suggested to indicate the presence of pre-fibrillar species. Celej *et al*.^50^ showed that pre-fibrillar oligomeric species of α-Synuclein were in an anti-parallel β-sheet orientation. These oligomers were believed to be cytotoxic based on the assembly of anti-parallel β-sheets into β-barrel-like structures that can potentially disrupt membranes^50^. The second-derivative Amide I band spectra clearly displayed a vibrational band centred at 1690 cm^−1^ (frequency limits 1685–1705 cm^−1^). This band is typically reported for anti-parallel β-sheet structure^51^, suggesting the presence of pre-fibrillar oligomeric species in this sample. Traces of this band also were observed for α-Synuclein fibrils. Sarroukh *et al*.^52^ estimated the percentage of anti-parallel and parallel organisation of β-strands by developing the β-sheet index, which they defined as the ratio of the intensities of the Amide I vibrational bands observed at 1695 cm^−1^ and 1630 cm^−1^. We estimated the β-indices (I_1695_/I_1630_) for α-Synuclein and the capped NAC 71–82 peptide (I_1690_/I_1627–1629_) using the spectra shown in Fig. 3, and the results were in agreement with previous reports on α-Synuclein fibrils produced after 72 h^50^. The final β-index value revealed the dominance of parallel β-sheet orientation over anti-parallel β-sheet orientation in this fibrillar system. The corresponding β-index for the capped NAC 71–82 peptide was higher for the initial solid state than for the fibrillar end-point, as expected, which reflected an anti-parallel to parallel β-sheet transformation. The capped NAC 71–82 peptide displayed more complete fibrillisation than the α-Synuclein protein, with fewer numbers of pre-fibrillar oligomeric species in anti-parallel β-sheet orientation. Since the non-capped NAC 71–82 peptide system did not exhibit a vibrational absorption peak at 1690 cm^−1^, a β-index was not calculated for this system.

A series of MD simulations was performed to obtain information about the molecular mechanisms involved in the initial phase of oligomerisation for the capped and non-capped NAC 71–82 peptides. These insights could potentially explain the structural differences in peptide fibrils observed during ATR FT-IR spectroscopy, such as the predominance of an anti-parallel pre-fibrillar oligomeric population of the capped NAC 71–82 peptide. After collecting a total of 20 μs of MD simulation data for both non-capped and capped NAC 71–82 peptides, initial calculations of the water solvation degree over time was performed to characterise the oligomerisation process. Our hypothesis was that formation of an aggregated hydrophobic core is driven by a desolvation mechanism (hydrophobic effect), which potentially could influence the final architecture of the pre-fibrillar oligomeric β-sheet structure. The discrepancy of water desolvation during oligomerisation of the capped and the non-capped NAC 71–82 peptides may affect their pre-fibrillar aggregate compactness differently, and thus could affect final fibril morphology.

**Figure 4:**
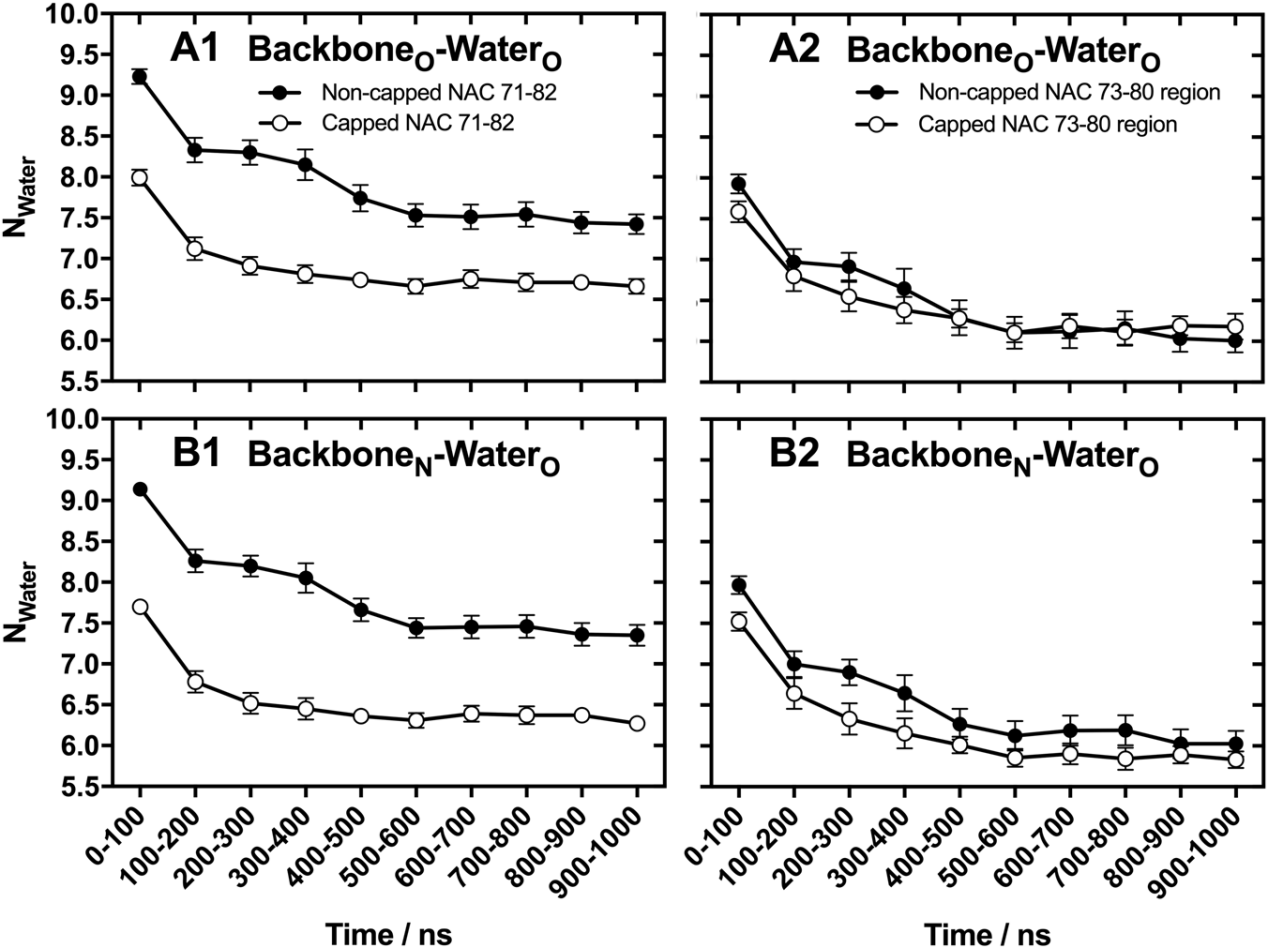
Estimation of the degree of water solvation calculated from MD-based peptide aggregation simulations. Calculated values (integration of the calculated RDFs) represent the average number of water molecules (N_Water_) within a 5.5-Å radius over 100-ns fragments of the total 1-μs simulation time of backbone carbonyl oxygen (Backbone_O_-Water_O_, *top*) (**A**) or a backbone amide nitrogen (Backbone_N_-Water_O_, *bottom*) (**B**) of the non-capped NAC 71–82 peptide + 0.15 M NaCl (filled circles) or the capped NAC 71–82 peptide (open circles). Values represent the mean ± standard error of the mean from 10 separate MD simulations of 1 μs each.

**Figure 5:**
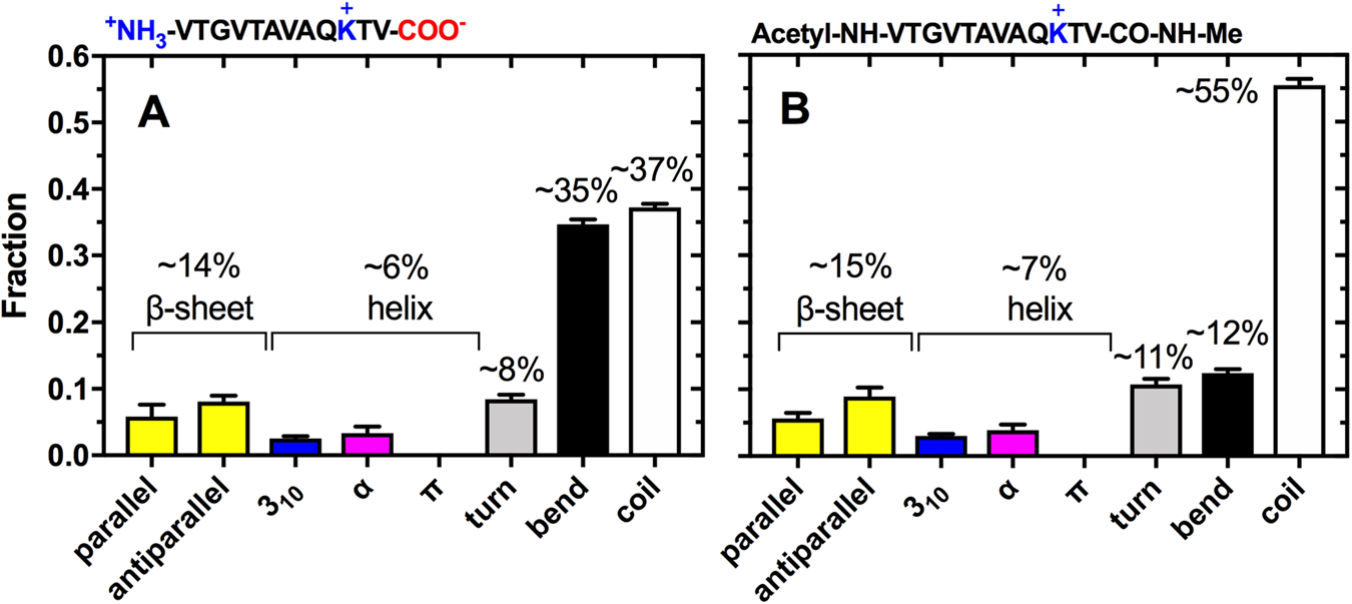
DSSP secondary structure analysis from MD trajectory data. Numbers describe the extent of secondary structure elements [parallel- and anti-parallel β-sheets, 3_10_-, α-, and π-helices, hydrogen-bonded turns, bends, and no secondary structure (coil)] that were populated during MD simulations of 10 copies of the non-capped NAC 71–82 peptide (in explicit water and 0.15 M NaCl) **(A)** and the capped NAC 71–82 peptide (in explicit water) **(B)**. The total occupancy of each type of secondary structure element is presented as a fraction of the total MD simulation time, and values are presented as mean ± standard error of the mean from 10 separate MD simulations of 1 μs each (Supplementary Figs S9 and S10).

Calculation of the average number of water molecules around each peptide backbone over time revealed that a water desolvation process was operating for both non-capped and capped NAC 71–82 peptides, which is a signature for oligomerisation (Fig. 4, and Supplementary Figs S5–S8 for single trajectory profiles). Closer inspection of the oligomerisation profiles suggested that the terminal charges of the non-capped NAC 71–82 peptide attracted more water than the peptide with acetylated and methyl amidated termini, which was determined after calculating RDFs for water accumulation around the backbone of the whole peptide (Fig.4A1,B1) and the NAC 73–80 region of both peptides (Fig. 4A2,B2). Secondary structure was analysed using the DSSP algorithm to evaluate the extent of β-sheet formation in the peptides during oligomerisation. Calculations of the average fraction of secondary structure elements populated revealed similar β-sheet, helical, and turn contents in the peptides (Fig. 5). Different peptide charge models can lead to differences in the modes by which they interact in monomeric form with macromolecular targets. Capping of the NAC 71–82 peptide may lead to more stable α-helical conformation than that of its charged counterpart. However, this topic was beyond the scope of this work.

The non-capped NAC 71–82 peptide had higher fraction of bend structure due to the presence of terminal charges. It is reasonable to assume that this results from water accumulation around these terminal groups, which restricts the conformational freedom of these peptide fragments.

**Figure 6:**
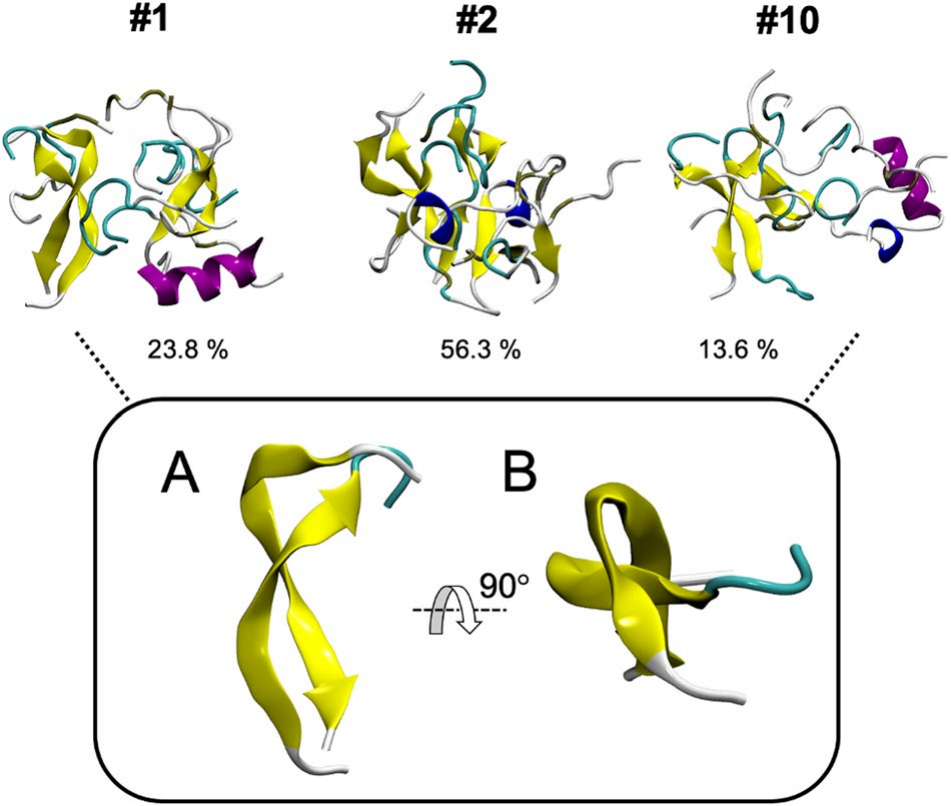
Selected top oligomeric cluster representatives of the capped NAC 71–82 peptide. The structures demonstrate evident anti-parallel twisted β-sheet motifs. Percentages written below each oligomeric peptide structure describe the time in cluster with respect to the total simulation time (#1, #2, and #10).

The structures populated during MD simulations were clustered using the DBScan algorithm to obtain quantitative information on oligomer stability and qualitative information on β-sheet architecture. Detailed analyses revealed a more stable fraction of oligomers (time in cluster with respect to the total simulation time) for the capped NAC 71–82 peptide than for oligomers of the non-capped NAC variant. The average fraction of stable oligomeric clusters for the capped and non-capped NAC 71–82 peptides was 60.0 ± 1.7% and 43.9 ± 2.5%, respectively (Supplementary Tables S2 and S3). The cluster representatives (the top three most-populated clusters for each peptide are presented in Supplementary Figs S11 and S12) revealed a twisted anti-parallel β-sheet motif in oligomeric clusters of the capped NAC 71–82 peptide, which was not detected in the non-capped NAC 71–82 peptide (Fig. 6). The twisted anti-parallel β-sheet motif is a common feature of β-hairpin structures that serve as fibrillar precursors in Alzheimer’s disease^53^ and type II diabetes^54^. The β-hairpin motif was previously reported to involve the 38–53 aa region and formed transiently during folding of full-length α-Synuclein^55^. A series of engineered proteins called β-wrapins was reported to bind to the 37–54 aa region of α-Synuclein, thereby reducing the fibrillisation rate of this protein^56^. Although these results suggest that the β-hairpin motif is important for the aggregation propensity of full-length protein, the role of the NAC 71–82 region in β-hairpin formation and interaction with macromolecular targets remains to be fully characterised.

Our MD and ATR FT-IR studies indicate that the average β-sheet structures populated revealed the co-existence of anti-parallel and parallel orientations of β-strands. However, results from MD simulations demonstrated a higher stability of anti-parallel β-sheets and more compact oligomeric structures in the capped NAC 71–82 peptide than in the non-capped NAC 71–82 peptide. The results suggest that the twisted anti-parallel β-sheet motif observed in the MD simulations could be associated with the pre-mature structure formed after a 72-h incubation, which was confirmed by an ATR FT-IR vibrational band at 1690 cm^−1^.

**Figure 7:**
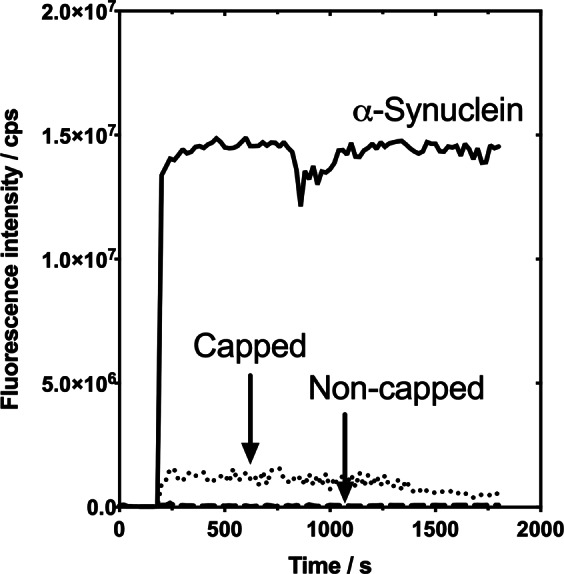
Time-based steady-state fluorescence analysis of ThT-fibril binding. Data of fluorescence emission over time of ThT after incubation with full-length α-Synuclein (black solid line), capped NAC 71–82 peptide (dotted line), and non-capped NAC 71–82 peptide (dashed line) aggregates.

To validate the β-sheet content, a ThT fluorescence binding assay was conducted after adding the dye to fibrillar samples that had been incubated for 72 h. The ThT steady-state fluorescence emission over time demonstrated that the fluorescence increased for α-Synuclein and capped NAC 71–82 fibrils, whereas the non-capped NAC 71–82 aggregated peptide was not observed to bind ThT (Fig. 7). ThT fluorescence increased approximately 10-fold more for α-Synuclein than for the capped NAC 71–82 fibrillar peptide, which may be due to the larger ThT binding capacity of these protein aggregates. Time correlated single photon counting (TCSPC) analyses of the α-Synuclein and capped NAC 71–82 aggregates provided additional details about the nature and extent of ThT binding to these fibrils (Table 1 and Supplementary Figs S13–S15). Two decay components were observed after incubation ThT with capped NAC 71–82 fibrils: the first component (A_1_) may be associated with an unbound state of ThT with a fluorescence lifetime of T_1_ ≈ 0.2 ns, whereas the second component (A_2_), suggested that 18% of the collected photons displayed a similar ThT fluorescence lifetime as that of the single lifetime observed for ThT incubated with α-Synuclein fibrils (T_1_ ≈ 1.9 ns).

**Table 1:** TCSPC data obtained from ThT-fibril binding experiments.

Fibril	Lifetimes/ns		Amplitudes/%		χ^2^
	T_1_	T_2_	A_1_	A_2_	
Capped NAC 71–82	0.203 ± 0.002	2.020 ± 0.002	82	18	1.17
α-Synuclein	1.915 ± 0.002		100		1.12
Non-capped NAC 71–82	≤0.100		100		19.6

The 1.9 ns lifetime for ThT bound to α-Synuclein fibrils was in agreement with a previous report of Sulatskaya and co-workers^57^. Only a single ThT lifetime of T_1_ < 0.1 ns (shorter than the detector resolution) was detected for ThT incubated with non-capped NAC 71–82 fibrils (Supplementary Fig. S14, which was expected based on the absence of ThT fluorescence at the emission wavelength.

To estimate the strength of ThT binding to capped NAC 71–82 fibrils, we performed a TCSPC assay where different concentrations of ThT (0–50 μM) were incubated with a constant concentration of fibril (0.1 mg · mL^−1^). The corresponding analysis of the fraction of bound ThT as a function of free unbound ThT concentration (T_1_ ≈ 0.2 ns) resulted in a saturation binding isotherm with the apparent dissociation constant K_D_ of 5 ± 3 μM (Supplementary Figs S17, S18, and Table S4). The control experiment varied the ThT concentration in the presence of a constant concentration of non-capped NAC 71–82 fibrils; the results did not identify the presence of a bound state of ThT, and did not demonstrate a concentration-dependent binding (Supplementary Fig. S16). Increasing the concentration of the non-capped NAC 71–82 fibrils from 0.1 to 0.2 mg · mL^−1^ increased the light scattering from aggregates (probably contributing to a higher amplitude of the T_1_ fluorescence life time).

The ThT-capped fibril binding affinity we observed was comparable with that reported by Ye *et al*.^58^ who performed a ThT fluorescence assay and reported a single class of ThT binding sites on α-Synuclein, with K_D_ = 0.588 ± 0.002 μM. A recent study suggested that there was an additional ThT binding site on α-Synuclein. Sulatskaya *et al*.^57^ performed equilibrium dialysis and reported a high-affinity binding mode with K_D_ in the micromolar range (the association binding constant K_A_ = 10^6^ M^−1^) and a weaker binding mode with K_D_ = 100 μM (the association binding constant K_A_ of 10^4^ M^−1^). Under the conditions employed in this work, the ThT binding affinity for the capped NAC 71–82 fibrils was similar to the average of the two modes (low- and high-affinity binding) of bound ThT to the full-length α-Synuclein protein. Based upon these observations, it can be concluded that the capped NAC 71–82 peptide fibrils bind ThT with a similar binding mode as that of α-Synuclein fibrils (high-affinity binding). This highlights the significance of the NAC 71–82 amino acid stretch on α-Synuclein fibrillisation and the use of a capped peptide model to capture the core β-sheet architecture of α-Synuclein fibrils.

**Figure 8:**
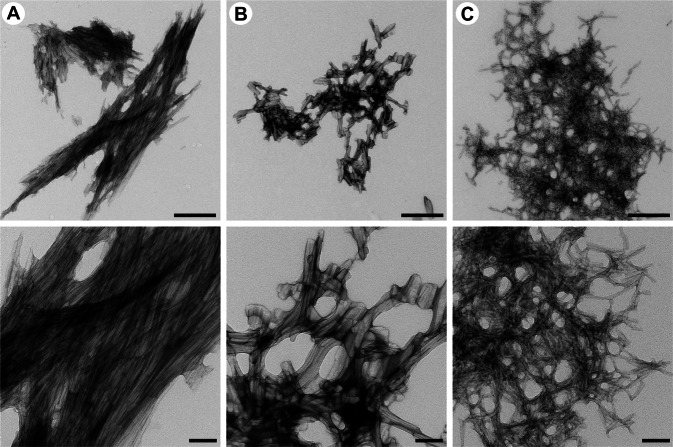
Transmission emission microscopy (TEM) analysis of fibrillar structure. Images of fibrillar structures of capped NAC 71–82 peptide (**A**), non-capped NAC 71–82 peptide (**B**), and α-Synuclein (**C**) after negative staining with 1.5% uranyl acetate. Scale bars represent 500 nm for upper panels and 100 nm for lower panels.

The final characterisation of end-point fibril morphology was performed using TEM. Non-capped NAC 71–82 fibrils displayed clumped fibrils with elongated tangles, in agreement with those obtained for α-Synuclein fibrils in this and previous work by Ariesandi *et al*.^59^ (Fig. 8). By contrast, capped NAC 71–82 fibrils did not display tangles but were rich in aggregated aligned fibrils in bundles (Fig. 8).

The unique fibril morphology observed for capped NAC 71–82 peptide may result from the additional cross-β structure that was identified by ATR FT-IR spectroscopy. Although the morphology of fibrils produced by the capped NAC 71–82 peptide is profoundly different from that of α-Synuclein fibrils, the data obtained from TEM analysis suggest the importance of the C-terminus, which was reported to govern the aggregation behaviour of the α-Synuclein protein. Rekas *et al*.^60^ demonstrated the chaperone activity of 96–140 aa region in the C-terminus, and suggested that the NAC 61–95 region contains a chaperone-binding site. Fibrils obtained from the non-capped NAC 71–82 peptide are morphologically more similar to α-Synuclein fibrils, probably due to the inclusion of charged termini, thereby modelling the charge balance of N-terminal (1–60 aa) and C-terminal (96–140 aa) domains of the full-length protein, which have net positive and negative charges, respectively.

Light scattering experiments revealed morphological differences between the non-capped and capped NAC 71–82 fibrils. The average fibril particle sizes were 3.3 μm and 4.6 μm for the capped and non-capped NAC 71–82 fibrils, respectively (Supplementary Fig. S19). The capped fibril particles had a narrower particle size distribution, thus reflecting a more homogenous particle size distribution. This observation demonstrated the differences in morphology of fibrils produced from the capped and non-capped NAC 71–82 peptide models. The larger fibril particles obtained for the non-capped NAC 71–82 peptide were consistent with the MD simulations conducted in this work that suggested increased water solvation around charged termini. Here, data derived from MD simulations representing the early stage of oligomerisation for both peptides provided valuable details about final fibril morphologies.

**Figure 9:**
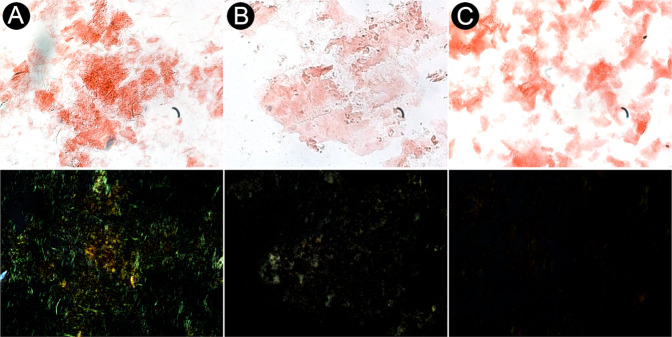
Brightfield and polarised microscopy images after Congo red staining of fibrils. Upper panels show staining of capped NAC 71–82 fibrils (**A**), non-capped NAC 71–82 fibrils (**B**), and α-Synuclein fibrils (**C**). Lower panels show apple-green birefringence in the corresponding samples. All images were captured at 20× magnification with a first-order red compensation filter for plane polarised light. Images were processed using Gimp 2.10 as described in the Methods section.

Congo red staining of fibrils was conducted to validate the presence of cross-β structure which has been proposed previously^61,62^. Brightfield microscopy revealed Congo red-positive staining for all samples (Fig. 9). The non-capped NAC 71–82 fibrils displayed less evident Congo red-positive staining than the capped NAC 71–82 peptide fibrils. To confirm true amyloid-like properties, samples were examined under polarised light to detect apple-green birefringence. The capped NAC 71–82 fibrils fulfilled all criteria and exhibited amyloid structure based on the results of Congo red staining^35,63^. By contrast, the α-Synuclein fibrils did not exhibit apple-green birefringence. To the best of our knowledge, no studies have reported that *in vitro*-generated or brain tissue-deposited α-Synuclein fibrils exhibit both positive Congo red staining and apple-green birefringence. We suggest that capped NAC 71–82 fibrils display apple-green birefringence due to the presence of an additional cross-β structure in these fibrils, which is not present in α-Synuclein fibrils. It should however be mentioned that the thickness of a stained sample may affect the detection of apple-green birefringence and lead to false positives or false negatives^64^. Cheng *et al*.^65^ used a Fmoc-VLK(Boc) tri-peptide to prepare fibrils that exhibited apple-green birefringence. The authors argued that this was due to a tightly packed cross-β structure, which supports our hypothesis. The observed differences in the Congo red staining and apple-green birefringence of capped and non-capped NAC 71–82 fibrils suggests a profound structural difference between these fibrils, and stresses the importance of using a capped NAC 71–82 peptide to model amyloid-forming propensity of the NAC core of full-length α-Synuclein. As a negative control for nonspecific binding of the Congo red dye, staining of Bovine Serum Albumin yielded a negative result (Supplementary Fig. S20).

## Conclusion

We investigated the NAC 71–82 amino acid stretch as the amyloid nucleus of α-Synuclein. ATR FT-IR analysis indicated the presence of a vibrational frequency band centred at 1619 cm^−1^ together with a lower frequency Amide I band at 1613 cm^−1^ for the capped NAC 71–82 fibrils, with the latter indicating a highly ordered cross-β structures that reflected the type of non-branched aggregated aligned fibrils in bundles that were observed in TEM analysis. Fibrils produced from the capped NAC 71–82 peptide showed greater binding of Thioflavin-T (ThT) than non-capped NAC 71–82 peptide, and fulfilled both Congo red-positive and apple-green birefringence amyloid detection criteria. Fluorescence lifetime analysis revealed a lifetime decay for ThT bound to capped NAC 71–82 fibrils of ~2.0 ns, in agreement with that observed for ThT-α-Synuclein fibril binding, thereby suggesting similar β-sheet binding of ThT for the two fibrillar samples. All-atom MD simulations representing early stages of peptide oligomerisation revealed water desolvation for both non-capped and capped NAC 71–82 peptides during the aggregation process. Although both peptides formed oligomers, the capped NAC 71–82 peptide formed more stable oligomers than the non-capped peptide. The increased fraction of the bend secondary structure element in non-capped NAC 71–82 peptide oligomers explained the absence of ThT binding with this peptide. Extraction of cluster representatives of NAC 71–82 oligomers revealed the presence of a stable, anti-parallel, twisted β-sheet motif in these structures. This feature may be the basis for the additional cross-β structure observed in capped NAC 71–82 fibrils, although further studies are needed. Finally, TEM analysis revealed a higher aggregation propensity to form aggregated aligned fibrils in bundles for the capped NAC 71–82 fibrils than the non-capped NAC 71–82- and α-Synuclein fibrils, which displayed clumped fibrils with elongated tangles. Particle size determination revealed that the capped NAC 71–82 peptide displayed smaller fibrils and a more homogenous population of particle sizes than the non-capped NAC 71–82 peptide.

These results are summarised in Table 2. They indicate that the amyloid cross-β structure for capped NAC 71–82 fibrils may constitute a central compact core of α-Synuclein aggregates. Fibrillisation of the capped NAC 71–82 peptide resulted in an additional cross-β structure that was previously reported in prion proteins. Our combined results shed light on the aggregation propensity of the NAC 71–82 amino acid stretch of α-Synuclein and the roles of the N- and C-terminal domains in regulating the rate of amyloid formation and final fibril morphology. Finally, we demonstrated that early aggregated forms of the capped NAC 71–82 peptide model yield structures that include anti-parallel and twisted β-sheet motifs. Due to the expected toxicity of these early aggregated forms, this β-sheet motif could be a promising molecular target for developing therapeutic strategies for PD and DLB.

**Table 2:** Data obtained from the spectroscopic and microscopic techniques employed that was used to assign the prepared fibrils as amyloids (Yes or No).

Analysis method	Full-length α-Synuclein	Non-capped NAC 71–82	Capped NAC 71–82
ThT binding	Yes	No	Yes
Congo red staining	Yes	No	Yes
Apple-green birefringence	No	No	Yes
FT-IR-spectroscopy	Yes	Yes	Yes

## Supplementary information

Supplementary Data

## Data Availability

All data generated or analysed during this study are included in this published article (and its Supplementary Information files).
