# Supplementary material for: Amyloid fibrils prepared using an acetylated and methyl amidated peptide model of the α-Synuclein NAC 71–82 amino acid stretch contain an additional cross-β structure also found in prion proteins
Source: Sci Rep. 2019 Nov 4;9:15949. doi: 10.1038/s41598-019-52206-5 (PMC6828723; doi:10.1038/s41598-019-52206-5)
Supplement: Supplementary file 1 — Supplementary Data [file 41598_2019_52206_MOESM1_ESM.pdf]

Supporting information for:

Amyloid fibrils prepared using an acetylated and methyl amidated peptide model of the α-Synuclein NAC 71-82 amino acid stretch contain an additional cross-β structure also found in prion proteins

Thomas Näsström[a]*, Per Ola Andersson[b,c], Christian Lejon[b] & Björn C.G. Karlsson[d]*

[a]Neurodegenerative Disorders Unit, Linnæus University, SE-392 31, Kalmar, Sweden. [b]FOI Swedish Defence Research Agency, CBRN Defence & Security, SE-901 82, Umeå, Sweden. [c]Department of Engineering Sciences: Applied Material Science, Uppsala University, SE-751 21, Uppsala, Sweden. [d]Physical Pharmacy Laboratory, Linnæus University Centre for Biomaterials Chemistry, Linnæus University, SE-392 31, Kalmar, Sweden.

Correspondence to:
* Björn C.G. Karlsson
E-mail: bjorn.karlsson@lnu.se
Tel: +46 480 446740

* Thomas Näsström
E-mail: thomas.nasstrom@lnu.se
Tel: +46 480 446329

Table of Contents

	Name	Page
**IN SILICO SYSTEMS STUDIED**	Table S1	S1
**RADIAL DISTRIBUTION FUNCTIONS (RDFs):**		
**Non-capped NAC 71-82 peptide + 0.15 M NaCl**		
- Backbone$_O$-Water$_O$	Figure S1	S2
- Backbone$_N$-Water$_O$	Figure S2	S3
**Capped NAC 71-82 peptide**		
- Backbone$_O$-Water$_O$	Figure S3	S4
- Backbone$_N$-Water$_O$	Figure S4	S5
**ATOMIC NUMBER DENSITIES:**		
**Non-capped NAC 71-82 peptide + 0.15 M NaCl**		
- Backbone$_O$-Water$_O$ & Backbone$_N$-Water$_O$	Figure S5	S6
**Non-capped NAC 73-80 region**		
- Backbone$_O$-Water$_O$ & Backbone$_N$-Water$_O$	Figure S6	S7
**Capped NAC 71-82 peptide**		
- Backbone$_O$-Water$_O$ & Backbone$_N$-Water$_O$	Figure S7	S8
**Capped NAC 73-80 region**		
Backbone$_O$-Water$_O$ & Backbone$_N$-Water$_O$	Figure S8	S9
**DSSP SECONDARY STRUCTURE ASSIGNMENTS:**		
**Non-capped NAC 71-82 peptide + 0.15 M NaCl**	Figure S9	S10
**Capped NAC 71-82 peptide**	Figure S10	S10

DBSCAN CLUSTERING DATA:

Non-capped NAC 71-82 peptide + 0.15 M NaCl

- Identified clusters and their occupancy — Table S2 — S11
- Structures of the top three most stable clusters — Figure S11 — S12

Capped NAC 71-82 peptide

- Found clusters and their occupancies — Table S3 — S13
- Structures of the top three most stable clusters — Figure S12 — S14

TCSPC ANALYSIS OF ThT-FIBRIL BINDING:

ThT (10 μM) & fibril (0.2 mg · mL^{-1})

- Full-length α-Synuclein + 0.15 M NaCl — Figure S13 — S15
- Non-capped NAC 71-82 peptide + 0.15 M NaCl — Figure S14 — S15
- Capped NAC 71-82 peptide — Figure S15 — S16

ThT (0-50 μM) & fibril (0.1 mg · mL^{-1})

- Non-capped NAC 71-82 peptide + 0.15 M NaCl — Figure S16 — S16
- Capped NAC 71-82 peptide — Figure S17 — S17
- Extracted lifetimes — Table S4 — S17
- One-site saturation binding isotherm — Figure S18 — S17

NANOPHOX ANALYSIS OF FIBRIL PARTICLE SIZE — Figure S19 — S18

CONGO RED STAINING OF BOVINE SERUM ALBUMIN CONTROL — Figure S20 — S18

IN SILICO SYSTEMS STUDIED

Table S1. Systems studied by molecular dynamics (MD) simulations.

		Number of molecules					*Box dim. /Å			Start conc. /mM			**Box dim, /Å			Final conc. /mM		
NAC 71-82	Sim.	Pep	Water	Na^+	Cl^-	No. atoms	X_0	Y_0	Z_0	Na^+	Cl^-	Pep	X	Y	Z	Na^+	Cl^-	Pep
Capped	#1	10	10000	-	10	31850	83.9	83.5	83.6	-	28.3	28.3	68.8	68.5	68.5	-	51.4	51.4
	#2	10	10000	-	10	31850	83.5	83.5	83.5	-	28.5	28.5	68.7	68.7	68.7	-	51.3	51.3
	#3	10	10000	-	10	31850	83.5	83.5	83.5	-	28.5	28.5	68.6	68.6	68.6	-	51.4	51.4
	#4	10	10000	-	10	31850	83.5	84.3	83.5	-	28.2	28.2	68.4	69.0	68.4	-	51.4	51.4
	#5	10	10000	-	10	31850	83.5	83.5	83.5	-	28.5	28.5	68.6	68.6	68.6	-	51.4	51.4
	#6	10	10000	-	10	31850	83.5	83.5	83.5	-	28.5	28.5	68.7	68.7	68.7	-	51.3	51.3
	#7	10	10000	-	10	31850	83.5	83.5	83.5	-	28.5	28.5	68.6	68.6	68.6	-	51.4	51.4
	#8	10	10000	-	10	31850	83.5	83.5	83.5	-	28.5	28.5	68.6	68.6	68.6	-	51.3	51.3
	#9	10	10000	-	10	31850	83.8	83.5	83.5	-	28.4	28.4	68.8	68.6	68.6	-	51.2	51.2
	#10	10	10000	-	10	31850	83.5	83.5	84.1	-	28.3	28.3	68.5	68.5	68.9	-	51.4	51.4
Non-capped +	#1	10	10000	29	39	31818	87.5	87.5	87.5	71.8	96.5	24.7	68.5	68.5	68.5	149.8	201.4	51.6
0.15 M NaCl	#2	10	10000	29	39	31818	87.5	87.5	87.5	71.8	96.5	24.7	68.5	68.5	68.5	149.8	201.4	51.6
	#3	10	10000	29	39	31818	87.7	87.7	87.5	71.5	96.2	24.7	68.7	68.6	68.5	149.1	200.5	51.4
	#4	10	10000	29	39	31818	87.6	88.0	88.2	70.7	95.1	24.4	68.4	68.7	68.8	148.9	200.2	51.3
	#5	10	10000	29	39	31818	88.7	88.3	88.0	69.9	94.0	24.1	68.9	68.5	68.2	149.6	201.1	51.6
	#6	10	10000	29	39	31818	88.2	87.5	87.5	71.2	95.8	24.6	68.9	68.4	68.4	149.3	200.8	51.5
	#7	10	10000	29	39	31818	87.5	87.5	88.1	71.3	95.9	24.6	68.4	68.4	68.9	149.3	200.8	51.5
	#8	10	10000	29	39	31818	88.3	88.2	87.5	70.7	95.1	24.4	68.8	68.7	68.2	149.3	200.8	51.5
	#9	10	10000	29	39	31818	87.7	87.5	88.0	71.2	95.8	24.6	68.6	68.4	68.7	149.3	200.8	51.5
	#10	10	10000	29	39	31818	87.6	87.5	87.7	71.6	96.3	24.7	68.6	68.5	68.6	149.3	200.8	51.5

*XYZ-coordinates obtained after building system boxes with PACKMOL. ** final equilibrated XYZ coordinates after 1 μs of MD simulation.

RADIAL DISTRIBUTION FUNCTIONS (RDFs)

Non-capped NAC 71-82 peptide + 0.15 M NaCl

Backbone$_O$-Water$_O$

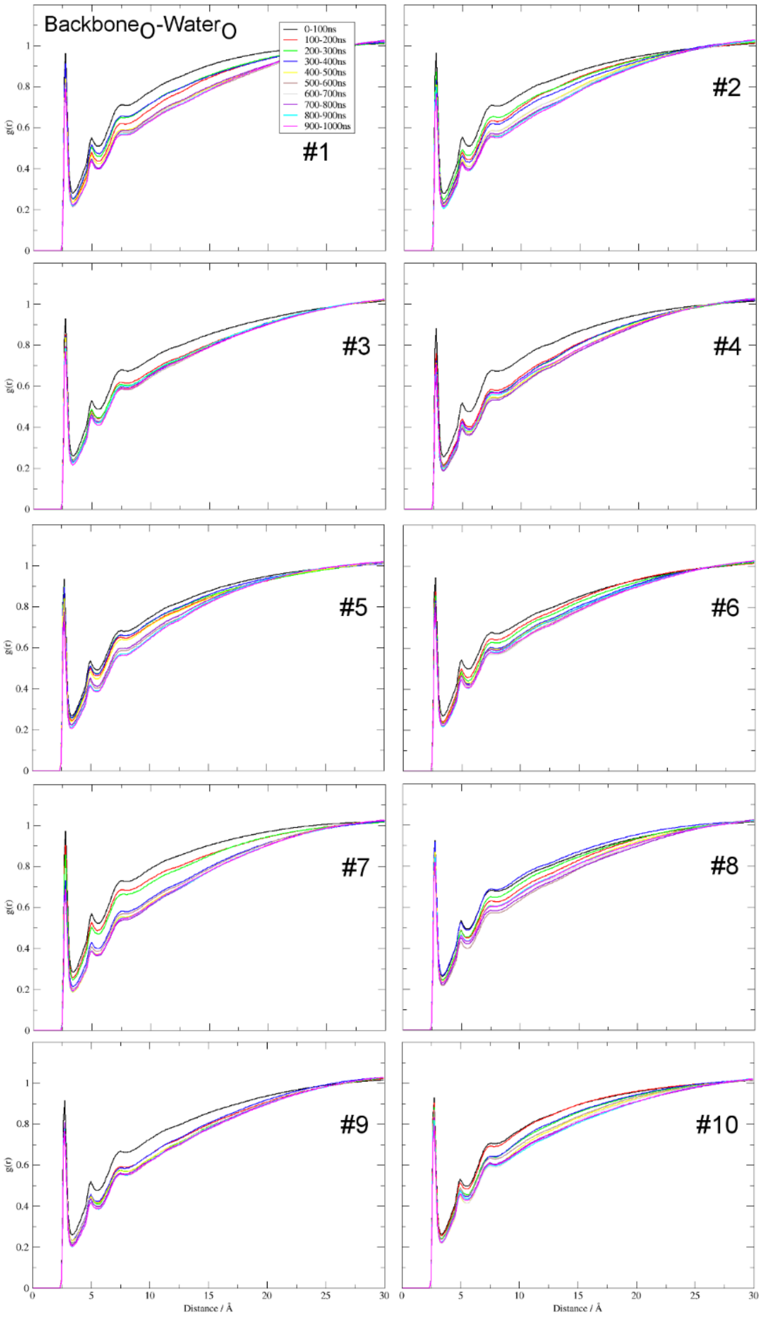

Figure S1. Non-capped NAC 71-82 peptide + 0.15 M NaCl Backbone$_O$-Water$_O$ RDFs calculated for each of the 10 (#1-#10) simulated replicas that are described in Table S1. Different colours in each panel represent RDFs that were calculated from 100 ns fragments over the total simulation time of 1 μs.

Backbone$_N$-Water$_O$

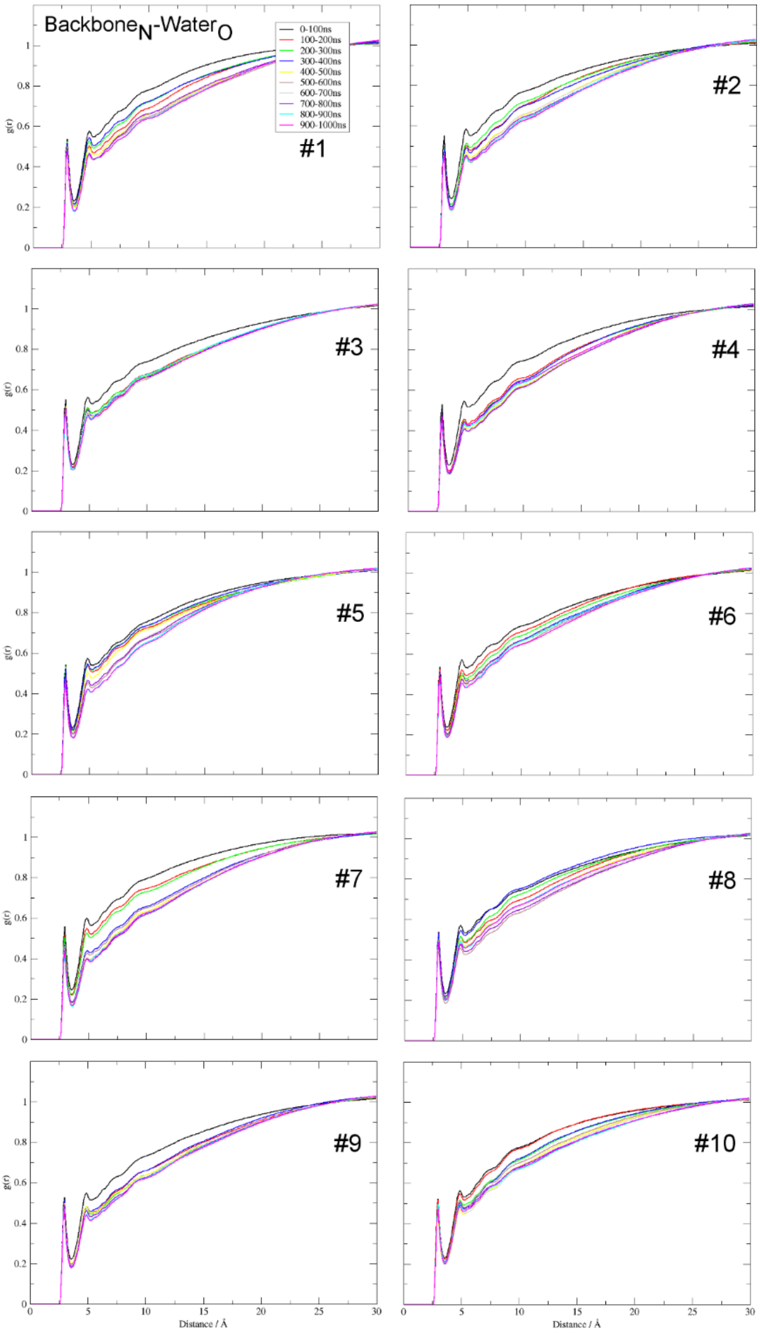

Figure S2. Non-capped NAC 71-82 peptide + 0.15 M NaCl Backbone$_N$-Water$_O$ RDFs calculated for each of the 10 (#1-#10) simulated replicas as described in Table S1. Different colours in each panel represent RDFs that were calculated from 100 ns fragments over the total simulation time of 1 μs.

Capped NAC 71-82 peptide

Backbone$_O$-Water$_O$

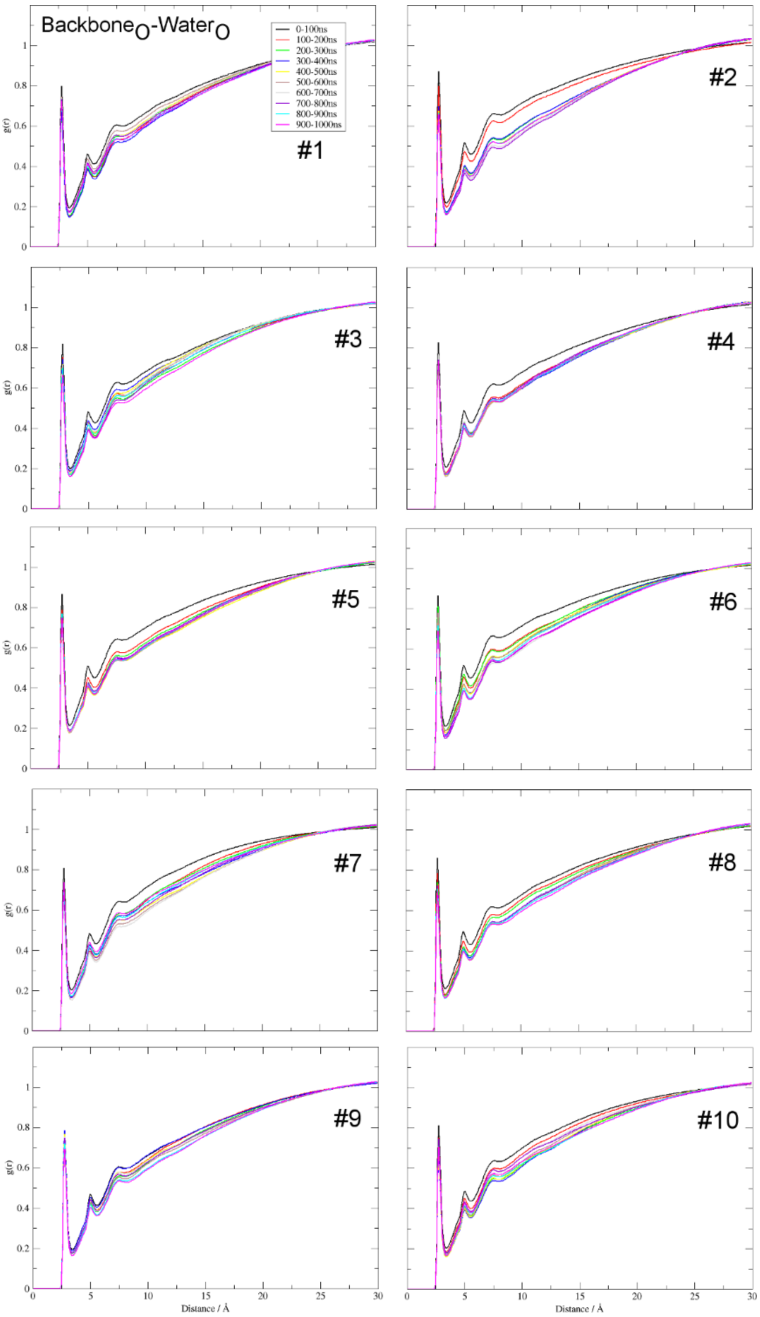

Figure S3. Capped NAC 71-82 peptide Backbone$_O$-Water$_O$ RDFs calculated for each of the 10 (#1-#10) simulated replicas that are described in Table S1. Different colours in each panel represent RDFs that were calculated from 100 ns fragments over the total simulation time of 1 μs.

Backbone$_N$-Water$_O$

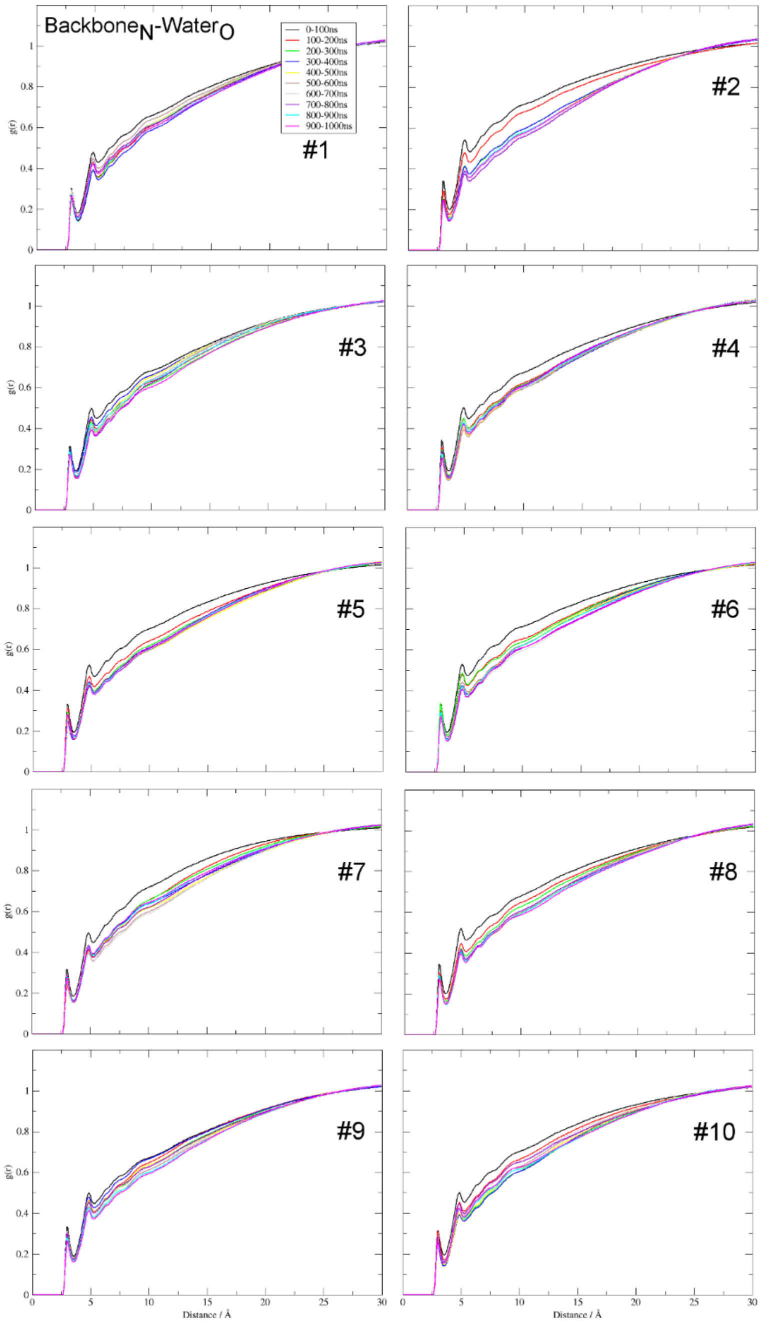

Figure S4. Capped NAC 71-82 peptide Backbone$_N$-Water$_O$ RDFs calculated for each of the 10 (#1-#10) simulated replicas as described in Table S1. Different colours in each panel represent RDFs that were calculated from 100 ns fragments over the total simulation time of 1 μs.

ATOMIC NUMBER DENSITIES

Non-capped NAC 71-82 peptide + 0.15 M NaCl

$Backbone_O$-$Water_O$ & $Backbone_N$-$Water_O$

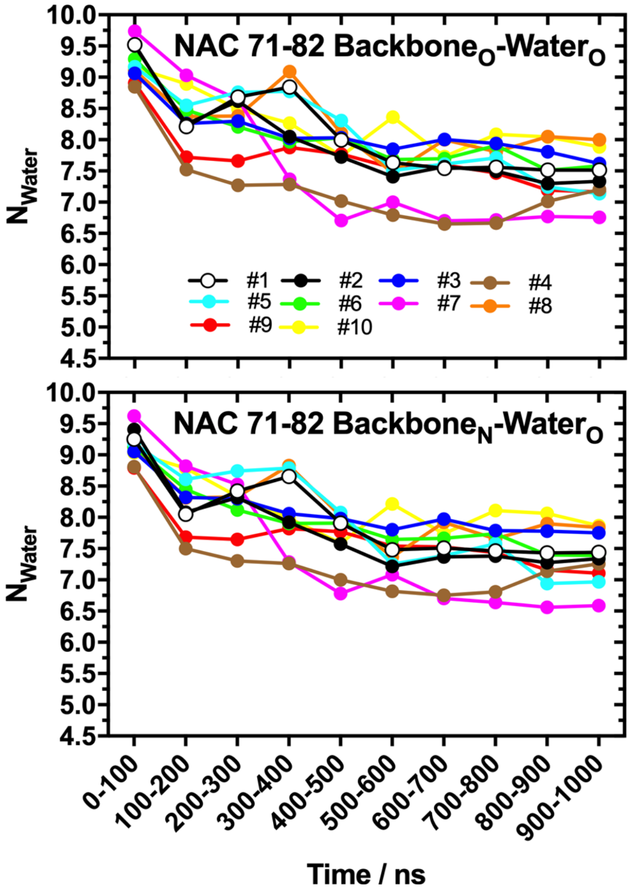

Figure S5. Calculated values (integration of the RDFs presented in Figs. S1 and S2) of the average number of water molecules found within 5.5 Å, from 100 ns fragments over the total simulation time of 1 µs, of the backbone carbonyl oxygen ($Backbone_O$-$Water_O$, *top*) or the backbone amide nitrogen ($Backbone_N$-$Water_O$, *bottom*) of the non-capped NAC 71-82 + 0.15 M NaCl peptide systems. Values presented are calculated from each of the 10 separate MD simulations (#1-#10) described in Table S1.

Non-capped NAC 73-80 region

Backbone$_O$-Water$_O$ & Backbone$_N$-Water$_O$

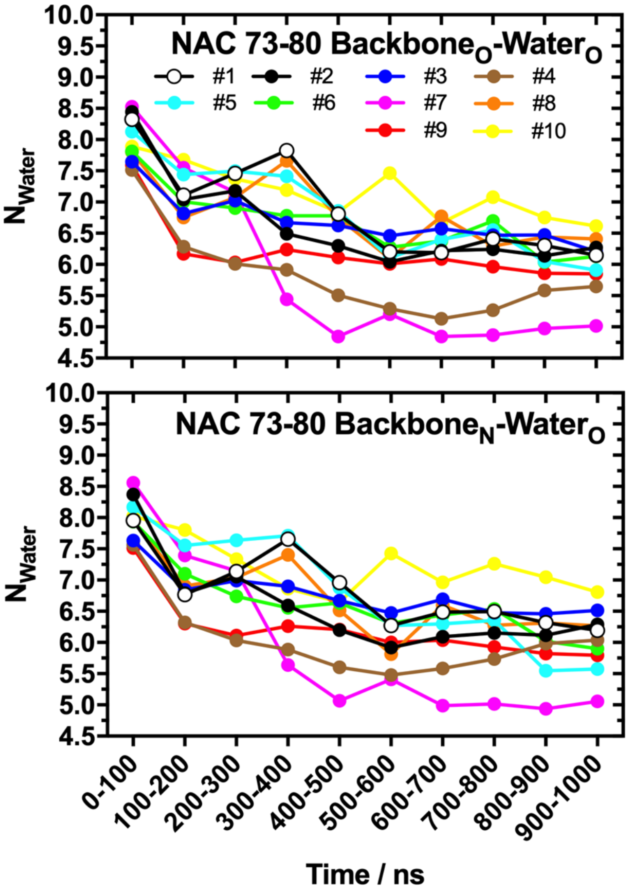

Figure S6. Calculated values (integration of calculated RDFs) of the average number of water molecules found within 5.5 Å, from 100 ns fragments over the total simulation time of 1 µs, of the backbone carbonyl oxygen (Backbone$_O$-Water$_O$, *top*) or the backbone amide nitrogen (Backbone$_N$-Water$_O$, *bottom*) of the non-capped NAC 73-80 region. Values presented are calculated from each of the 10 separate MD simulations (#1-#10) described in Table S1.

Capped NAC 71-82 peptide

Backbone$_O$-Water$_O$ & Backbone$_N$-Water$_O$

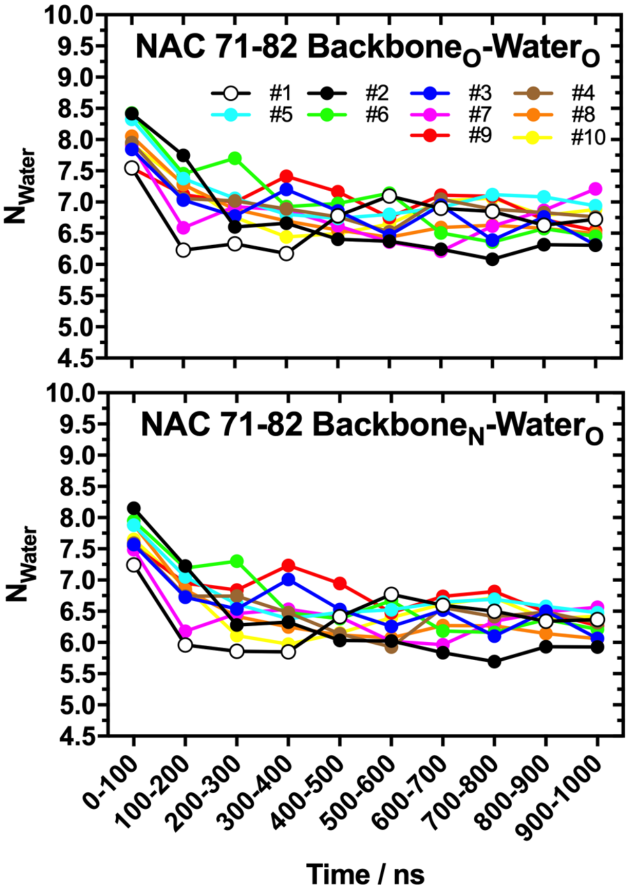

Figure S7. Calculated values (integration of the RDFs presented in Figs. S3 and S4) of the average number of water molecules found within 5.5 Å, over 100 ns fragments of the total simulation time of 1 μs, of the backbone carbonyl oxygen (Backbone$_O$-Water$_O$, *top*) or the backbone amide nitrogen (Backbone$_N$-Water$_O$, *bottom*) of the capped NAC 71-82 peptide systems. Values presented are calculated from each of the 10 separate MD simulations (#1-#10) described in Table S1.

Capped NAC 73-80 region

Backbone$_O$-Water$_O$ & Backbone$_N$-Water$_O$

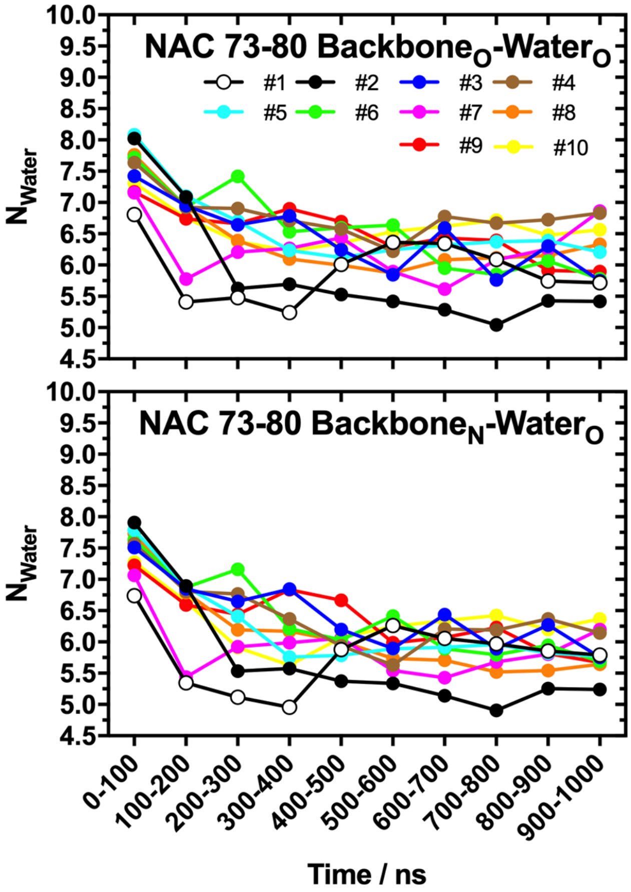

Figure S8. Calculated values (integration of calculated RDFs) of the average number of water molecules found within 5.5 Å, over 100 ns fragments of the total simulation time of 1 μs, of the backbone carbonyl oxygen (Backbone$_O$-Water$_O$, *top*) or the backbone amide nitrogen (Backbone$_N$-Water$_O$, *bottom*) of the Capped NAC 73-80 region. Values presented are calculated from each of the 10 separate MD simulations (#1-#10) described in Table S1.

DSSP SECONDARY STRUCTURE ASSIGNMENTS

Non-capped NAC 71-82 peptide + 0.15 M NaCl

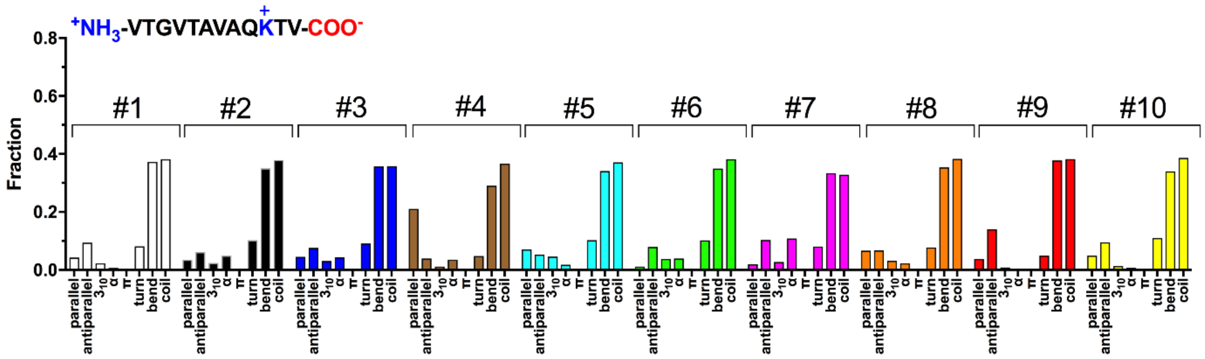

Figure S9. Values show the different DSSP secondary structure elements [parallel- and anti-parallel β-sheets, 3_{10}-, α-, and π-helices, hydrogen-bonded turns, bends, and no secondary structure (coil)] that were populated during MD simulations of 10 copies (#1-#10) of the non-capped NAC 71-82 peptide in explicit water with 0.15 M NaCl. The total occupancy of each type of secondary structure element is here presented as a fraction of the 1 µs MD simulation time for each replicate. Bars shown represent mean values.

Capped NAC 71-82 peptide

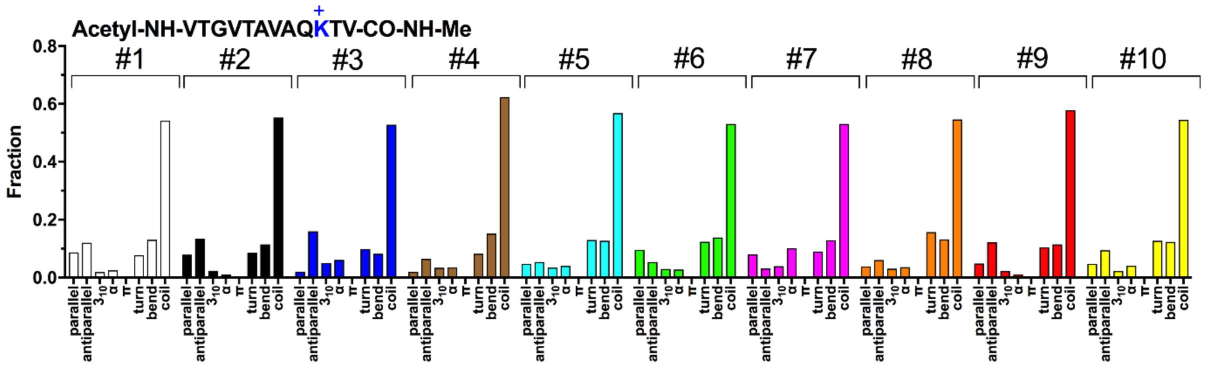

Figure S10. Values show the different secondary structure elements [parallel- and anti-parallel β-sheets, 3_{10}-, α-, and π-helices, hydrogen-bonded turns, bends, and no secondary structure (coil)] that were populated during MD simulations of 10 copies (#1-#10) of the capped NAC 71-82 peptide in explicit water. The total occupancy of each type of secondary structure element is here presented as a fraction of the 1 µs MD simulation time for each replicate. Bars shown represent mean values.

DBSCAN CLUSTERING DATA

Non-capped NAC 71-82 peptide + 0.15 M NaCl

Table S2. Data obtained from DBScan clustering of the non-capped NAC 71-82 peptide in the 10 systems studied by MD simulation (#1-#10).

	Fraction of the total simulation time									
Cluster	#1	#2	#3	#4	#5	#6	#7	#8	#9	#10
c0	0.110	0.076	0.060	0.344	0.143	0.112	0.316	0.065	0.250	0.058
c1	0.089	0.068	0.057	0.094	0.117	0.110	0.051	0.042	0.138	0.044
c2	0.051	0.048	0.051	0.035	0.033	0.033	0.039	0.034	0.119	0.044
c3	0.043	0.046	0.047	0.033	0.023	0.027	0.017	0.028	0.030	0.040
c4	0.038	0.040	0.038	0.009	0.017	0.026	0.010	0.020	0.011	0.036
c5	0.026	0.028	0.027	0.005	0.015	0.020	0.007	0.018	0.009	0.026
c6	0.012	0.013	0.021		0.008	0.018	0.004	0.016	0.006	0.026
c7	0.012	0.010	0.018		0.008	0.012	0.003	0.016	0.006	0.018
c8	0.009	0.010	0.017		0.006	0.010		0.014	0.005	0.016
c9	0.009	0.009	0.013		0.005	0.009		0.010	0.005	0.014
c10	0.006	0.008	0.012			0.008		0.010	0.005	0.009
c11	0.005	0.006	0.011			0.008		0.008		0.009
c12	0.005	0.005	0.011			0.007		0.007		0.009
c13	0.005	0.005	0.011			0.007		0.007		0.007
c14	0.004	0.004	0.011			0.007		0.006		0.006
c15		0.004	0.010			0.006		0.006		0.006
c16			0.010			0.006		0.005		0.006
c17			0.009			0.004		0.005		0.005
c18			0.008			0.004		0.004		0.005
c19			0.008			0.004				0.005
c20			0.008			0.004				
c21			0.007			0.002				
c22			0.007							
c23			0.006							
c24			0.005							
c25			0.005							
c26			0.005							
c27			0.005							
c28			0.004							
c29			0.004							
c30			0.004							
**SUM**	0.42	0.38	0.51	0.52	0.38	0.44	0.45	0.32	0.58	0.39

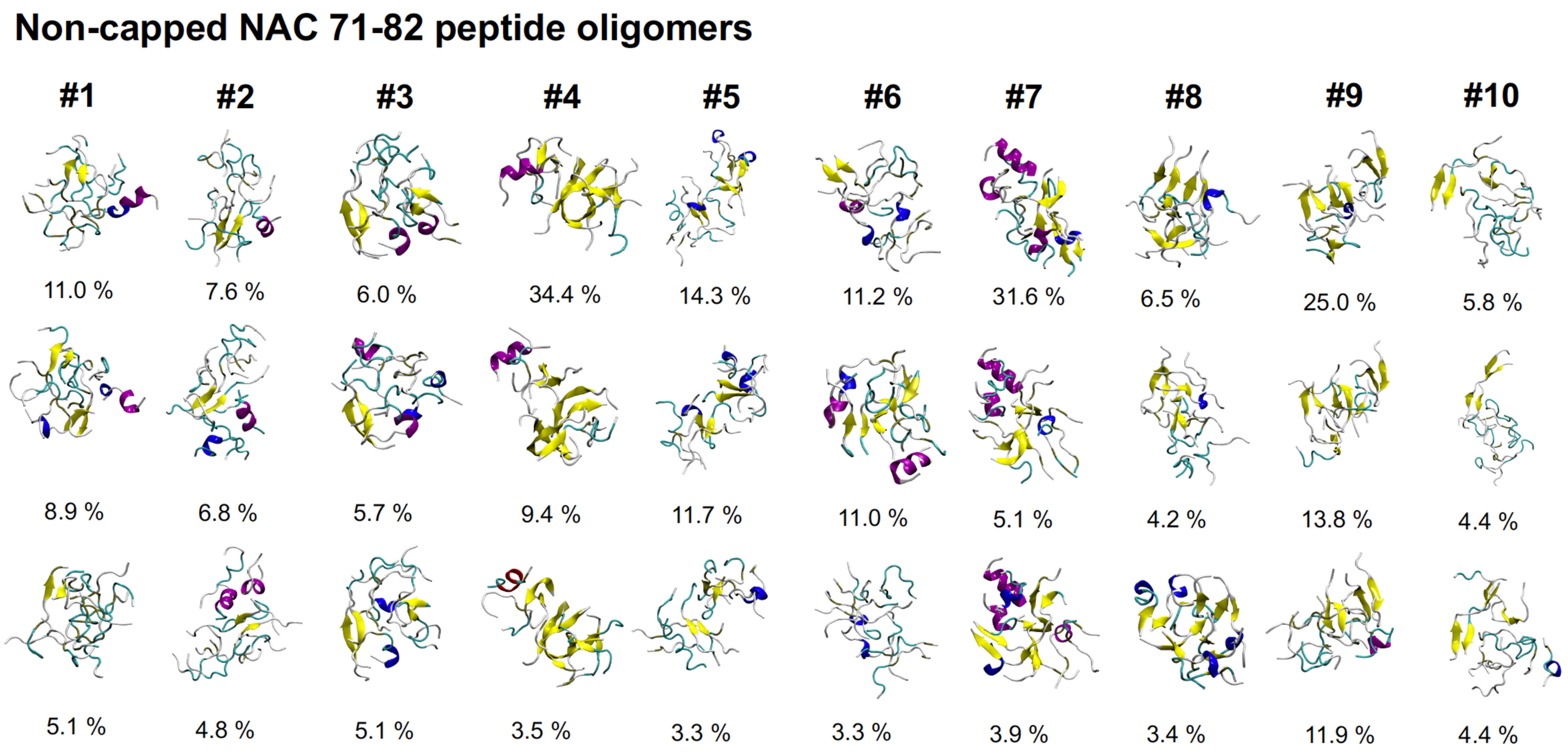

Figure S11. The top three most stable non-capped NAC 71-82 peptide oligomers that were identified after performing DBScan clustering of the structures populated (presented in Table S2) in each of the 10 separate MD simulations (#1-#10) that were conducted. Secondary structure elements presented were derived using the STRIDE algorithm (Frishman, D. & Argos, P. Knowledge-Based Secondary Structure Assignment. *Proteins: Struct., Funct., Genet.* **23**, 566-579 (1995)) which is implemented in the VMD software (v. 1.9.1., University of Illinois at Urbana-Champaign, USA) (Humphrey, W., Dalke, A. & Schulten, K. VMD - Visual Molecular Dynamics. *J. Molec. Graphics* **14**, 33-38 (1996).

Capped NAC 71-82 Peptide

Table S3. Data obtained from DBScan clustering of the capped NAC 71-82 peptide in the 10 systems studied by MD simulation (#1-#10).

	Fraction of the total simulation time									
Cluster	#1	#2	#3	#4	#5	#6	#7	#8	#9	#10
c0	0.238	0.563	0.189	0.239	0.271	0.159	0.179	0.235	0.112	0.136
c1	0.170	0.012	0.058	0.091	0.100	0.153	0.047	0.171	0.083	0.093
c2	0.073	0.006	0.043	0.091	0.060	0.074	0.031	0.090	0.060	0.089
c3	0.041		0.041	0.064	0.057	0.063	0.021	0.088	0.035	0.063
c4	0.037		0.035	0.044	0.029	0.034	0.021	0.016	0.021	0.040
c5	0.036		0.028	0.037	0.024	0.025	0.020	0.015	0.020	0.039
c6	0.012		0.017	0.021	0.021	0.021	0.018	0.009	0.015	0.029
c7	0.010		0.014	0.017	0.018	0.018	0.015	0.009	0.012	0.026
c8	0.008		0.013	0.015	0.013	0.016	0.014	0.007	0.011	0.024
c9	0.007		0.012	0.006	0.009	0.016	0.014	0.006	0.010	0.009
c10	0.005		0.009	0.005	0.008	0.012	0.014	0.005	0.010	0.007
c11	0.005		0.007	0.005	0.008	0.011	0.012		0.010	0.006
c12	0.005		0.007	0.005	0.007	0.011	0.012		0.010	0.005
c13	0.005		0.007		0.006	0.010	0.011		0.009	0.005
c14	0.005		0.006		0.005	0.007	0.010		0.008	0.005
c15	0.004		0.006		0.004	0.006	0.010		0.008	0.004
c16			0.006		0.004	0.005	0.009		0.007	0.004
c17			0.006		0.003		0.009		0.007	
c18			0.006				0.008		0.007	
c19			0.005				0.008		0.007	
c20			0.005				0.008		0.006	
c21			0.005				0.007		0.006	
c22			0.004				0.006		0.006	
c23			0.004				0.006		0.006	
c24			0.004				0.006		0.005	
c25			0.004				0.006		0.005	
c26			0.004				0.005		0.004	
c27							0.005		0.004	
c28							0.005		0.003	
c29							0.005			
c30							0.005			
**SUM**	0.66	0.58	0.55	0.64	0.65	0.64	0.55	0.65	0.51	0.58

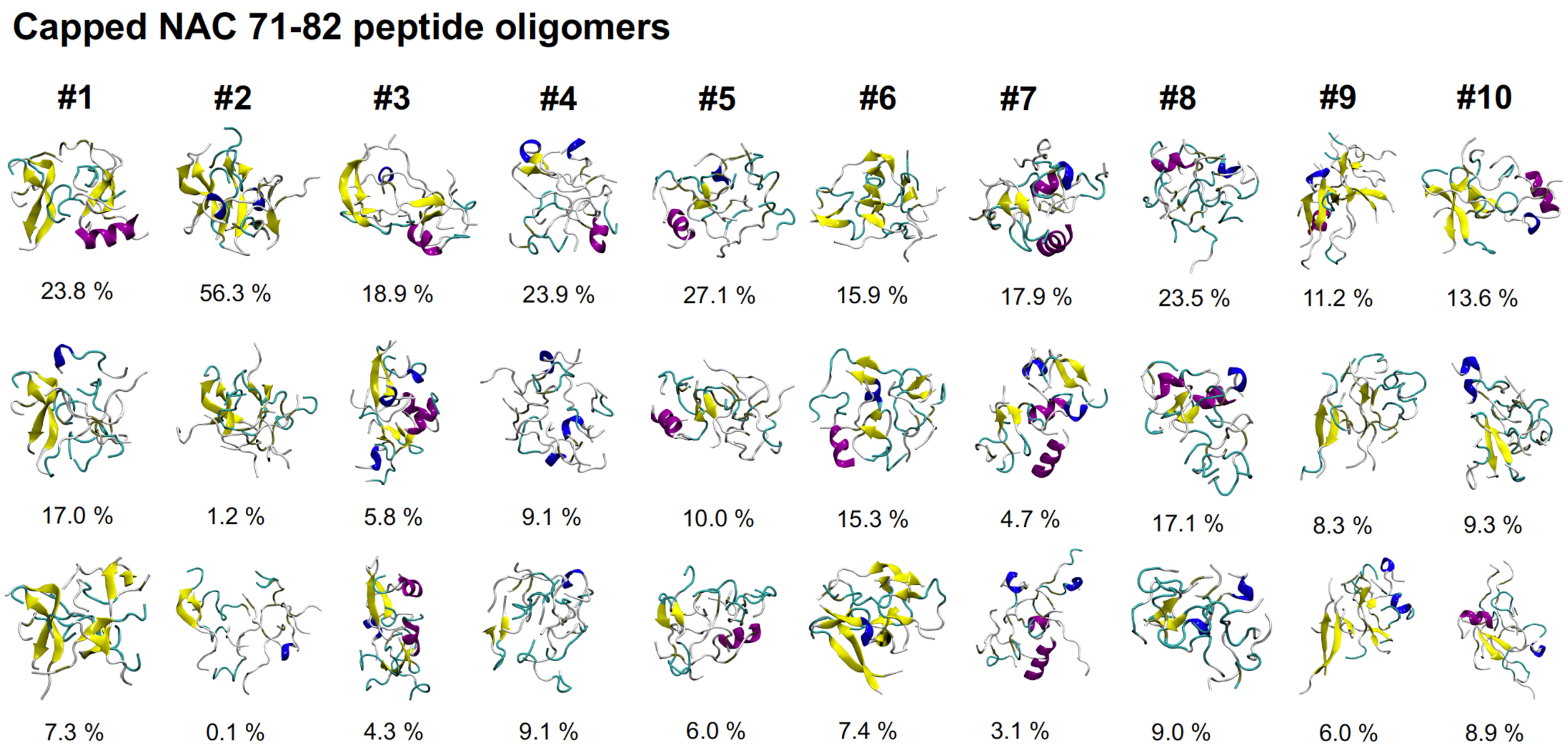

Figure S12. The top three most stable capped NAC 71-82 peptide oligomers that were identified after performing DBScan clustering of the structures populated (presented in Table S3) in each of the 10 separate MD simulations (#1-#10) that were conducted. Secondary structure elements presented were derived using the STRIDE algorithm (Frishman, D. & Argos, P. Knowledge-Based Secondary Structure Assignment. *Proteins: Struct., Funct., Genet.* **23**, 566-579 (1995)) which is implemented in the VMD software (v. 1.9.1., University of Illinois at Urbana-Champaign, USA) (Humphrey, W., Dalke, A. & Schulten, K. VMD - Visual Molecular Dynamics. *J. Molec. Graphics* **14**, 33-38 (1996)).

TCSPC ANALYSIS OF ThT-FIBRIL BINDING

ThT (10 μM) & fibril (0.2 mg · mL^{-1})

Full-length α-Synuclein + 0.15 M NaCl

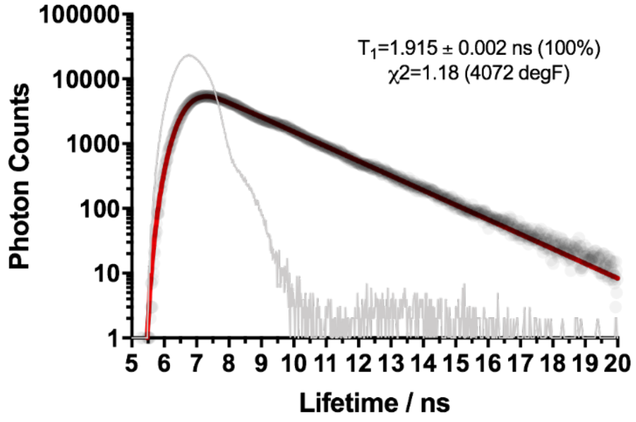

Figure S13. Instrument response function (LUDOX®, grey), ThT (10 μM) fluorescence in the presence of 0.2 mg · mL^{-1} of full-length α-Synuclein fibrils, and the fitted double exponential decay data (red).

Non-capped NAC 71-82 peptide + 0.15 M NaCl

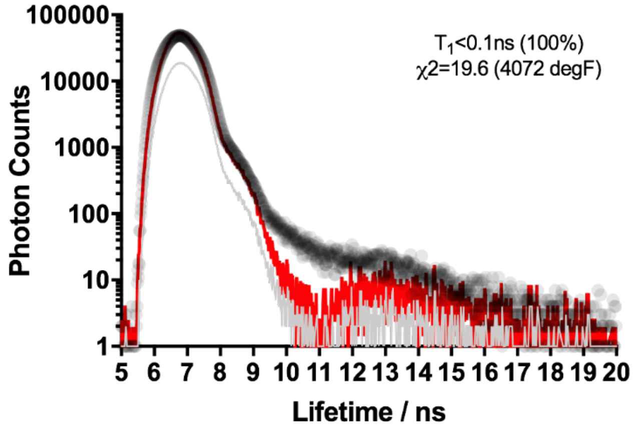

Figure S14. Instrument response function (LUDOX®, grey), ThT (10 μM) fluorescence in the presence of 0.2 mg · mL^{-1} of non-capped NAC 71-82 peptide + 0.15 M NaCl fibrils, and the fitted single exponential decay data (red).

Capped NAC 71-82 peptide

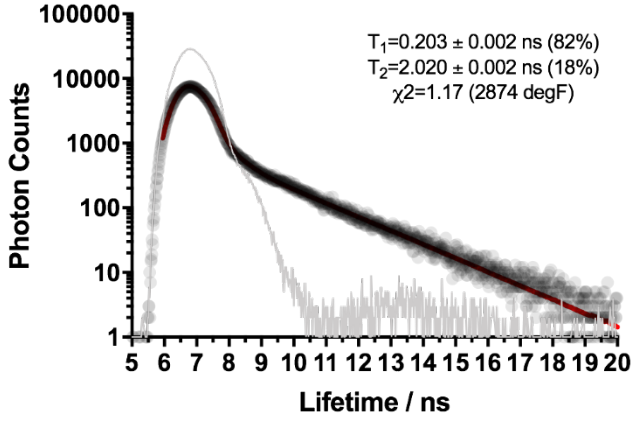

Figure S15. Instrument response function (LUDOX®, grey), ThT (10 μM) fluorescence in the presence of 0.2 mg · mL^{-1} of capped NAC 71-82 peptide fibrils, and the fitted double exponential decay data (red).

ThT (0-50 μM) & fibril (0.1 mg · mL^{-1})

Non-capped NAC 71-82 peptide + 0.15 M NaCl

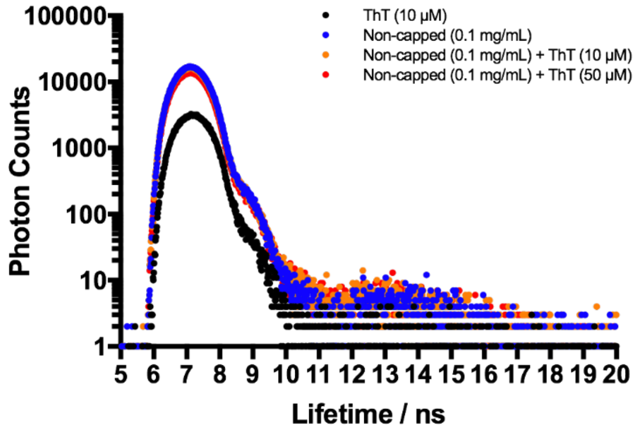

Figure S16. Photon histograms from varying concentrations of ThT [0 μM (blue), 10 μM (orange), and 50 μM (red)] in the presence of 0.1 mg · mL^{-1} of non-capped NAC 71-82 peptide + 0.15 M NaCl fibrils. A control decay profile for ThT (10 μM) in the absence of fibrils is included (black).

Capped NAC 71-82 peptide

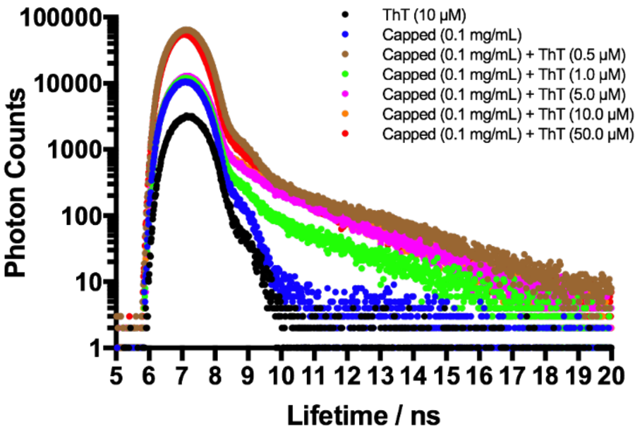

Figure S17. Photon histograms from varying concentrations of ThT [0 µM (blue), 0.5 µM (brown), 1.0 µM (green), 5.0 µM (purple), 10 µM (orange), and 50 µM (red)] in the presence of 0.1 mg · mL^{-1} of Capped NAC 71-82 peptide fibrils. A control decay profile for ThT (10 µM) in the absence of fibrils is included (black).

Extracted Lifetimes

Table S4. TCSPC ThT (0.5-50 µM) and capped NAC 71-82 fibrils (0.1 mg · mL^{-1}) binding data.

	Lifetimes / ns		Amplitudes / %		
[ThT] / µM	T_1	T_2	A_1	A_2	χ^2
0.5	< 0.10 (0.079 ± 0.002)	2.39 ± 0.02	97.37 ± 0.03	2.63 ± 0.03	1.19
1.0	0.103 ± 0.003	2.23 ± 0.04	95.93 ± 0.01	4.07 ± 0.02	1.20
5.0	0.105 ± 0.003	2.13 ± 0.01	89.71 ± 0.01	10.29 ± 0.02	1.15
10	0.104 ± 0.004	2.04 ± 0.01	87.70 ± 0.01	12.34 ± 0.02	1.16
50	0.100 ± 0.002	2.19 ± 0.02	97.35 ± 0.03	2.65 ± 0.03	1.88

One-site saturation binding isotherm

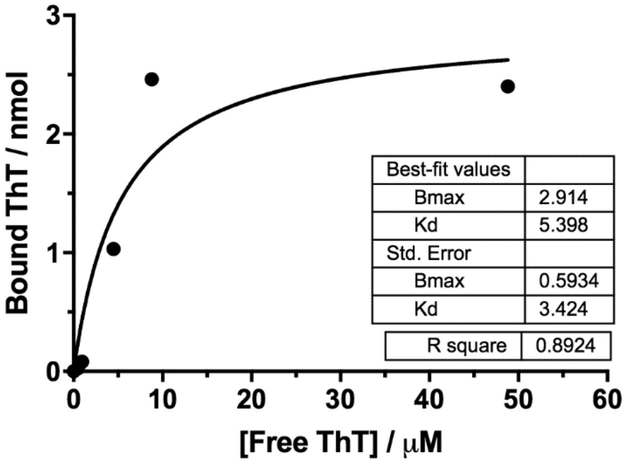

Figure S18. A one-site saturation binding isotherm constructed from the change of the amplitude (A_2) and the lifetime (T_2) as a function of the concentration of free unbound ThT, [free ThT].

NANOPHOX ANALYSIS OF FIBRIL PARTICLE SIZE

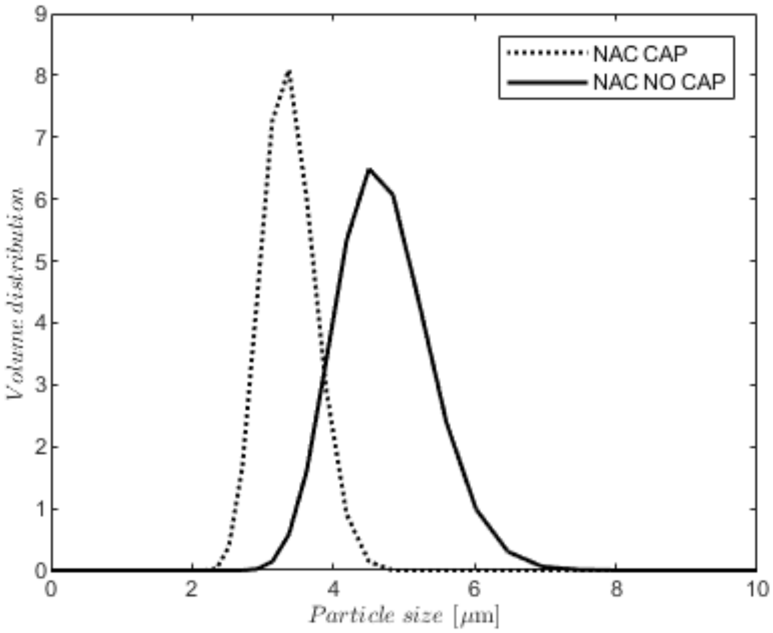

Figure S19. Particle size distribution by volume as measured by photon cross correlation spectroscopy. Non-capped NAC 71-82 peptide + 0.15 M NaCl fibrils (solid line) and capped NAC 71-82 peptide fibrils (dotted line) have a clear aggregation tendency, with the capped fragment displaying smaller fibril size (median 3.3 μm) with a narrower volumetric distribution than the non-capped NAC 71-82 peptide size (median 4.6 μm).

CONGO RED STAINING OF BOVINE SERUM ALBUMIN CONTROL

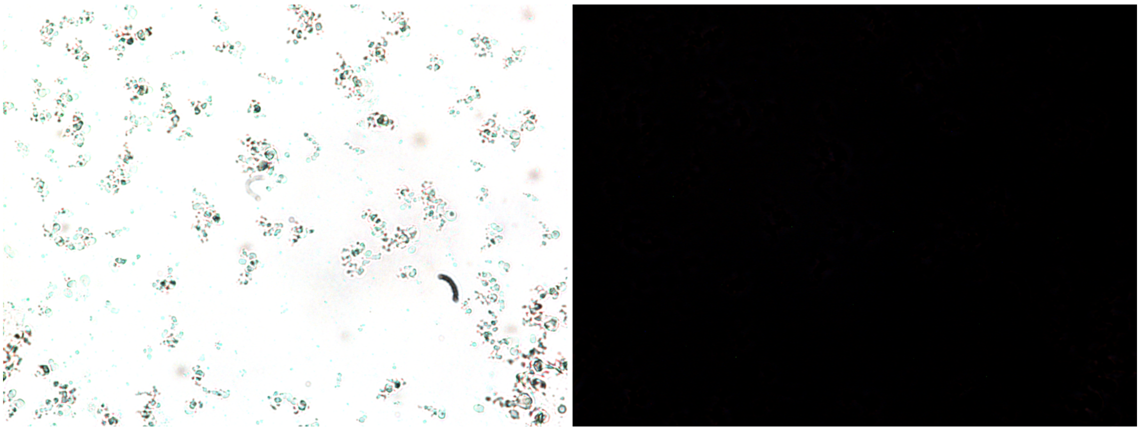

Figure S20. Images of Congo red stained Bovine Serum Albumin. Brightfield microscopy (*left*) and apple-green birefringence (*right*). Pictures were captured at 20× magnification using a with a first order red compensation filter for plane polarised light. Acquired images were processed using the Gimp software as being described in the Methods section.
